# Novel insights into chloroplast genome evolution in the green macroalgal genus *Ulva* (Ulvophyceae, Chlorophyta)

**DOI:** 10.3389/fpls.2023.1126175

**Published:** 2023-04-18

**Authors:** Feng Liu, Nansheng Chen, Hongshu Wang, Jiamin Li, Jing Wang, Fan Qu

**Affiliations:** ^1^ CAS Key Laboratory of Marine Ecology and Environmental Sciences, Institute of Oceanology, Chinese Academy of Sciences (IOCAS), Qingdao, Shandong, China; ^2^ Marine Ecology and Environmental Science Laboratory, Pilot National Laboratory for Marine Science and Technology (Qingdao), Qingdao, Shandong, China; ^3^ Center for Ocean Mega-Science, Chinese Academy of Sciences, Qingdao, Shandong, China; ^4^ Harbin University of Science and Technology, Weihai, Shandong, China

**Keywords:** chloroplast genome, Ulvophyceae, comparative genomics, GC content, group I/II intron, genome rearrangement

## Abstract

To understand the evolutionary driving forces of chloroplast (or plastid) genomes (plastomes) in the green macroalgal genus *Ulva* (Ulvophyceae, Chlorophyta), in this study, we sequenced and constructed seven complete chloroplast genomes from five *Ulva* species, and conducted comparative genomic analysis of *Ulva* plastomes in Ulvophyceae. *Ulva* plastome evolution reflects the strong selection pressure driving the compactness of genome organization and the decrease of overall GC composition. The overall plastome sequences including canonical genes, introns, derived foreign sequences and non-coding regions show a synergetic decrease in GC content at varying degrees. Fast degeneration of plastome sequences including non-core genes (*minD* and *trnR3*), derived foreign sequences, and noncoding spacer regions was accompanied by the marked decrease of their GC composition. Plastome introns preferentially resided in conserved housekeeping genes with high GC content and long length, as might be related to high GC content of target site sequences recognized by intron-encoded proteins (IEPs), and to more target sites contained by long GC-rich genes. Many foreign DNA sequences integrated into different intergenic regions contain some homologous specific *orf*s with high similarity, indicating that they could have been derived from the same origin. The invasion of foreign sequences seems to be an important driving force for plastome rearrangement in these IR-lacking *Ulva* cpDNAs. Gene partitioning pattern has changed and distribution range of gene clusters has expanded after the loss of IR, indicating that genome rearrangement was more extensive and more frequent in *Ulva* plastomes, which was markedly different from that in IR-containing ulvophycean plastomes. These new insights greatly enhance our understanding of plastome evolution in ecologically important *Ulva* seaweeds.

## Introduction

The green algal class Ulvophyceae harbors at least 13 orders and more than 2700 species thus far, and ranks second in the number of species among Chlorophyta only next to the class Chlorophyceae (Guiry and Guiry, 2023). Species in the Ulvophyceae show great diversification of cytological types and morphological complexity, which varied from small unicellular species (e.g. Scotinosphaerales), to large multicellular thalli composed of uninucleate cells (e.g. Ulvales) or multinucleate cells (e.g. Cladophorales), to the gigantic single-celled coenocytic thalli (e.g. Bryopsidales and Dasycladales) (Cocquyt et al., 2010; Leliaert et al., 2012; Gulbrandsen et al., 2021). Meanwhile, their chloroplast or plastid genomes (plastomes, or cpDNAs, or ptDNAs) display miraculous variations in genome architecture, genome size, GC content, gene density, intron content and gene order (Lang and Nedelcu, 2012; Smith, 2017; Turmel and Lemieux, 2018), ranging from the circular 195.9-kb plastome with two inverted repeats (IRs) in *Pseudendoclonium akinetum* (Ulotrichales) (Pombert et al., 2005), which is the first ulvophycean cpDNA sequenced, to the 34 multiple hairpin cpDNA chromosomes in *Boodlea composita* (Cladophorales) with high GC content (average 57%) (Del Cortona et al., 2017), from the compact 74.5-kb IR-lacking plastome in *Callipsygma wilsonis* (Bryopsidales) (Cremen et al., 2018) to the approximately 2000-kb plastome in *Acetabularia acetabulum* (Dasycladales) with a high noncoding content (more than 85%) (de Vries et al., 2013).

The green macroalgal genus *Ulva* Linnaeus 1753 (Ulvophyceae, Chlorophyta) is the species-richest genus in the Ulvales. As more *Ulva* species have been accurately identified recently, 102 species names have been flagged as accepted taxonomically up to now (Guiry and Guiry, 2023). Globally, many *Ulva* seaweeds (e.g. *Ulva prolifera*, *Ulva compressa*, and *Ulva meridionalis*) are notorious for their rapid vegetative growth in eutrophic waters, leading to green tides formed by the accumulation of excess biomass (Wang et al., 2019; Liu et al., 2022b). *Ulva* simple morphologies show high similarity at the interspecific level, meanwhile cytological and morphological features could vary greatly at the intraspecific level, thus accurate identification of *Ulva* species has been challenging (e.g. Blomster et al., 2002; Hayden and Waaland, 2002). The use of molecular markers (e.g., ITS, *rbcL*, and *tufA*) for species identification has become the mainstream method to ensure the accuracy and credibility of identification results (e.g. Hofmann et al., 2010; Hughey et al., 2019; Steinhagen et al., 2019). However, due to the limited differentiation signals of these marker sequences, their resolution is inadequate for identifying closely related *Ulva* species. Organelle genomes (cpDNAs and mtDNAs) as super molecular markers have been proved to be powerful to understand the evolution and molecular species concepts in the genus *Ulva*, and are potential resources for developing specific high-resolution molecular markers (Mitsuhashi et al., 2020). Recently, phylogenomic analysis based on organelle genome data clearly depicted the evolutionary nature of double crown radiation in the phylogeny and speciation of *Ulva* species (Liu and Melton, 2021; Liu et al., 2022a; Liu et al., 2022b).

The data of *Ulva* plastomes have accumulated rapidly recently based on efficient high-throughput sequencing technology (Melton et al., 2015; Fort et al., 2021; Hughey et al., 2021), which makes it possible for more accurately understanding of the evolution trend of *Ulva* plastomes on a more detailed and specific sampling scale. A total of 33 plastomes from 17 *Ulva* species have been documented in the GenBank database thus far. The sequenced *Ulva* plastomes show many unique features when compared with the counterparts in other ulvophycean lineages. These *Ulva* plastomes belong to the compact circular IR-lacking plastomes with the smaller size (86.73 - 119.87 kb) and the lowest GC content (23.89 - 26.25%) within the Ulvophyceae (**Table 1**). Variations in the *Ulva* plastome size at interspecific and intraspecific level were mainly caused by differences in content of group I/II introns, integration of foreign DNA fragments and content of intergenic regions (Liu and Melton, 2021). The *Ulva* plastomes show high conservation in repertoire of canonical genes, and share the same set of 100 core genes including 71 protein-coding genes (PCGs), three ribosomal RNA (rRNA) genes and 26 transfer RNA (tRNA) genes (Wang et al., 2021). The organelle division inhibitor factor gene, *minD*, was observed to be present only in the plastome of *Ulva aragoënsis* (Liu and Melton, 2021), which used to be regarded as *Ulva flexuosa* (Cai et al., 2017). Only one group II (derived) intron (intron *infA*-62) were shared by all sequenced *Ulva* plastomes, and all other introns displayed highly variable and sporadic distribution pattern. *Ulva* plastome architectures were dynamic and plastic not conserved at the intrageneric level due to frequent genome rearrangements (Mitsuhashi et al., 2020; Liu and Melton, 2021).

**Table 1:** The sequenced 40 plastomes of *Ulva* species for comparative analysis.

Lineage	Subclade	Species	Abbr.	Accession number	Size (bp)	GC (%)	References
*Ulva* I	IA	*Ulva prolifera*	*Upr1*	OP985129	93,066	24.78	This study
		*Ulva prolifera*	*Upr2*	OP985130	93,066	24.78	This study
		*Ulva prolifera*	*Upr3*	OP985131	93,072	24.78	This study
		*Ulva prolifera*	*Upr4*	KX342867	93,066	24.78	Jiang et al., 2019
		*Ulva prolifera*	*Upr5*	MZ571508	99,724	25.28	GenBank
		*Ulva linza*	*Uli*	KX058323	86,726	24.79	Wang et al., 2017
		*Ulva torta*	*Uto1*	OL684342	112,034	24.89	This study
		*Ulva torta*	*Uto2*	MZ703011	105,423	25.24	Wen et al., 2022
		*Ulva californica*	*Uca*	MZ561475	92,126	24.71	Lin et al., 2022
		*Ulva aragoënsis*	*Uar1*	OP985132	87,172	24.68	This study
		*Ulva aragoënsis* (Ulva flexuosa*)	*Uar2*	KX579943	89,414	24.97	Cai et al., 2017
	IB	*Ulva gigantea*	*Ugi*	MT179350	117,606	25.73	Fort et al., 2021
		*Ulva lactuca* (syn. *Ulva fasciata*)	*Ula1*	KT882614	96,005	24.87	Melton and Lopez-Bautista, 2017
		*Ulva lactuca*	*Ula2*	MH730972	95,997	24.87	Hughey et al., 2019
		*Ulva ohnoi*	*Uoh*	AP018696	103,313	25.44	Suzuki et al., 2018
		*Ulva lacinulata* (Ulva laetevirens*)	*Ulc1*	MT179351	103,444	25.40	Fort et al., 2021
		*Ulva lacinulata*	*Ulc2*	MW543061	107,242	25.82	Hughey et al., 2021
		*Ulva lacinulata* (Ulva laetevirens*)	*Ulc3*	MW531676	110,889	25.63	Wang et al., 2021
		*Ulva lacinulata* (Ulva rigida*)	*Ulc4*	MN389525	103,523	25.40	Hughey et al., 2021
		*Ulva* sp. A AF-2021 (Ulva rigida*)	*Usp2*	MT179352	96,673	24.57	Fort et al., 2021
	IC	*Ulva meridionalis*	*Ume*	OP985133	122,172	24.86	This study
		*Ulva* sp. UNA00071828	*Usp1*	KP720616	99,983	25.30	Melton et al., 2015
		*Ulva tepida*	*Ute*	OL684341	94,449	24.49	This study
		*Ulva* sp. Q253	*Usp3*	MW699788	88,801**	23.89	GenBank
		*Ulva* sp. (Ulva prolifera*)	*Usp3*	MN853879	88,801	23.89	GenBank
		*Ulva* sp. (Ulva meridionalis*)	*Usp3*	MN889540	88,653	23.91	Liu J. et al., 2020
*Ulva* II	IIA	*Ulva compressa*	*Uco1*	MW548841	114,291	26.23	Liu and Melton, 2021
		*Ulva compressa*	*Uco2*	MW344287	91,189	25.86	Liu and Melton, 2021
		*Ulva compressa*	*Uco3*	MW353781	96,824	26.17	Liu and Melton, 2021
		*Ulva compressa* (syn. *Ulva mutabilis*)	*Uco4*	MK069584	119,866	26.24	GenBank
		*Ulva compressa*	*Uco5*	MK069585	>89,164	26.25	GenBank
		*Ulva compressa*	*Uco6*	MT916929	94,226	25.80	Xia et al., 2021
		*Ulva compressa*	*Uco7*	KX595275	96,808	26.18	GenBank
		*Ulva intestinalis*	*Uin*	MZ158703	99,041	24.97	Wang et al., 2021
	IIB	*Ulva rigida* (Ulva rotundata*)	*Uri1*	MT179353	118,206	26.12	Fort et al., 2021
		*Ulva rigida*	*Uri2*	MW543060	117,995	26.13	Hughey et al., 2021
		*Ulva fenestrata*	*Ufe*	MT179349	94,654	25.27	Fort et al., 2021
		*Ulva australis* (syn. *Ulva pertusa*)	*Uau1*	MN853875	104,380	25.66	Han et al., 2020
		*Ulva australis*	*Uau2*	LC507117	102,899	25.33	Mitsuhashi et al., 2020
		*Ulva australis*	*Uau3*	MT179348	99,820**	25.21	Fort et al., 2021

* The *Ulva* plastomes with wrong species name assignment, which were deposited in the GenBank database, have been corrected. *Ulva laetevirens* (MT179351), *Ulva rigida* (MT179352), and *Ulva rotundata* (MT179353) have been corrected to *Ulva lacinulata* (MT179351), *Ulva* sp. A AF-2021 (MT179352), and *Ulva rigida* (MT179353), respectively (Fort et al., 2021).

In this study, we sequenced and constructed seven complete plastomes from five *Ulva* species including *Ulva prolifera* O.F.Müller, *Ulva aragoënsis* (Bliding) Maggs, *Ulva torta* (Mertens) Trevisan, *Ulva tepida* Y. Masakiyo & S. Shimada, and *Ulva meridionalis* R. Horimoto & S. Shimada, and conducted comparative plastomic analysis to understand the evolutionary driving forces in ecologically important *Ulva* seaweeds.

## Materials and methods

### Sample collection and DNA extraction

Three free-floating algal samples of *Ulva prolifera* O.F.Müller (LF001, LF002 and LF003) were collected on 2 Jul. 2021 at the First (N36°05’53’’, E120°34’17’’), Second (N36°04′96″, E120°34′83″) and Third (N36°05′03″, E120°36′82″) bathing beaches along the coast of Qingdao, Shandong, China, respectively. The sessile samples of *Ulva aragoënsis* (Bliding) Maggs (LF005) and *Ulva tepida* Y.Masakiyo & S.Shimada (LF006) were collected on 11 Aug. 2021 at the Trestle Bridge (N36°06′09″, E120°31′08″) and the First bathing beach (N36°05′34″, E120°33′81″) along the coast of Qingdao, Shandong, China, respectively. The free-floating algal thalli of *Ulva torta* (Mertens) Trevisan (LF007) and *Ulva meridionalis* R.Horimoto & S.Shimada (LF010) were sampled on 4 Aug. 2021 in the Sakura Lake (37°07’30’’-56’’N, 122°27’03’’-50’’E), Rongcheng, Shandong, China. These *Ulva* samples were stored in coolers (5-8°C) after collection and transported back to the laboratory within 48 hours. Algal thallus for each individual *Ulva* thallus was cultured in a 9-cm diameter Petri dish containing 25-mL L1 medium with 0.5‰ GeO_2_, 50 µg/mL Dipterex (Fengcheng Animal Medicine Co., Ltd, China) and a suite of antibiotics (per mL: 50 µg streptomycin, 66.6 µg gentamycin, 20 µg ciprofloxacin, 2.2 µg chloramphenicol, and 100 µg ampicillin) (Shibl et al., 2020). The culture was maintained at 18°C, 100 - 120 μmol photons m^-2^ s^-1^ in the photoperiod of 12 h light: 12 h darkness in a GXZ-380C temperature-controlled incubator (Ningbo Jiangnan, China). After at least one week of culture, fresh algal tissue from each *Ulva* thallus was used for DNA extraction using a Plant Genome DNA Kit (DP305, Tiangen Biotech, Beijing, China) according to the manufacturer’s instructions. Species identification was conducted based on phylogenetic analyses of two common DNA marker datasets, including the nuclear ITS region including the 5.8S rDNA gene, and the chloroplast *rbcL* gene (Hayden and Waaland, 2002; Liu F. et al., 2020).

### DNA sequencing and plastome assembly

The quality and concentration of total genomic DNA extracted were checked using a NanoPhotometer spectrophotometer (Implen, CA, USA), and a Qubit 2.0 Flurometer (Life Technologies, CA, USA), respectively. Qualified DNA samples were fragmented into 350 bp by Covaris S220 ultrasonic crater for library construction. The libraries were sequenced on an Illumina NovaSeq platform (Illumina, USA) using paired-end sequencing, yielding about 10 Gb sequencing raw data of paired-end reads with 150 bp in length for each *Ulva* sample. Clean data were harvested by trimming sequencing adapters and removing short or low-quality reads from the raw data. Complete *Ulva* plastomes were constructed by the GetOrganelle v1.7.1 (Jin et al., 2020). The plastome of *U. compressa* (MW353781) was used as the reference genome for assembly. Plastome assemblies were re-examined by aligning reads against the assembled plastome sequence using the MEM algorithm of BWA v0.7.17 (Li and Durbin, 2010). VarScan v2.3.9 (Koboldt et al., 2009) and IGV v2.8.12 (Robinson et al., 2011) were employed to examine mutation sites and to verify assembly results, respectively.

### Annotation of *Ulva* plastomes

Protein-coding genes (PCGs) were annotated by using the Open Reading Frame Finder at the National Center for Biotechnology Information (NCBI) website (https://www.ncbi.nlm.nih.gov/orffinder/), and by aligning homologous PCGs from *Ulva* cpDNAs deposited in the GenBank database with the newly sequenced *Ulva* plastomes. Transfer RNA genes (tRNAs) were searched for by reconstructing their cloverleaf structures using the tRNAscan-SE 2.0 software with default parameters (Chan et al., 2021). Ribosomal RNA genes (rRNAs) were identified by using the RNAweasel (https://megasun.bch.umontreal.ca/apps/rnaweasel/), and by aligning homologous rRNAs. The free-standing and intronic open reading frames (*orf*s) were found by using the Open Reading Frame Finder at the NCBI website. Intron insertion-sites were determined manually by aligning the intron-containing homologous genes, and corresponding genes in the *U. compressa* (MW353781) plastome were used as a reference (Liu and Melton, 2021). Intron name was defined as host gene plus insertion site. The class and core structure of all these introns were determined by using the RNAweasel and Mfold (Zuker, 2003). The core domains of intron-encoded proteins (IEPs) and free-standing specific ORFs were determined by significant Pfam-A matches (Bateman et al., 2000). To ensure the accuracy of comparative analysis, we have re-annotated the plastomes of *Ulva* species and *Blidingia minima* (MK408749 and MT948112) deposited in the GenBank database with the same method as above. All annotation results (including genes and introns) were manually verified. In some *Ulva* plastomes (e.g. MZ561475, MK069585, MT916929, and KX342867), incorrect annotations and abnormal sequence errors have been corrected in our subsequent comparative analysis.

### Plastome comparison and phylogenetic analysis

Base composition of *Ulva* plastomes and other DNA sequences was determined by using MEGA 7.0 (Kumar et al., 2016). Tandem repeats were analyzed by using Tandem Repeats Finder with parameter settings of two for matches and seven for mismatches and indels (Benson, 1999). Differences and identity values of DNA sequences were calculated by use of BioEdit v7.1.9 (Hall, 1999). Synteny analysis of *Ulva* plastomes was executed by using Mauve v2.3.1 software with default parameters (Darling et al., 2010). A new class of specific *orf*s named Ucp-*orf* was found in *Ulva* plastomes. The thorough search for Ucp-*orf*-like sequences in *Ulva* plastomes was conducted against the NCBI nucleotide database. A total of 29 full-length Ucp-*orf*s were detected in 17 of 40 *Ulva* cpDNAs. Multiple sequence alignments of Ucp-ORFs were conducted by using ClustalX 1.83 with the default settings (Thompson et al., 1997). The structural domain or motif in Ucp-ORFs was searched on the HMMER website (https://www.ebi.ac.uk/Tools/hmmer/search/phmmer) and using InterProScan tool (Paysan-Lafosse et al., 2023). The phylogenetic relationships were inferred with the Maximum Likelihood (ML) method based on the JTT matrix-based model (Jones et al., 1992) by using MEGA 7.0 (Kumar et al., 2016). There was a total of 113 positions in the final dataset of Ucp-ORFs. Phylogenomic trees were constructed based on two plastome datasets including nucleotide (nt) sequences of 100 common genes and amino acid (aa) sequences of 71 common PCGs from 40 *Ulva*. The nt sequences of 100 genes and the aa sequences of 71 PCGs were individually aligned, checked and concatenated using ClustalX 1.83 with default settings (Thompson et al., 1997). Maximum-likelihood trees were constructed by IQ-TREE (Trifinopoulos et al., 2016) using default parameters with 1000 ultrafast bootstrap analysis (Minh et al., 2013). The substitution model conducted by IQ-TREE was GTR+G for nt dataset and cpREV+F+I+G4 for aa dataset, respectively. *Blidingia minima* (MK408749 and MT948112) was used as the outgroup.

## Results and discussion

### *Ulva* plastomes show a clear evolutionary trend of becoming smaller and more compact

These seven newly obtained plastomes from five *Ulva* species were successfully assembled as circular-mapping molecules, with sizes ranging from 87.2 kb in *U. aragoënsis* (*Uar1*) to 122.2 kb in *U. meridionalis* (*Ume*) (**Table 1**). The 122.2-kb cpDNA of *Ume* is the largest *Ulva* plastome sequenced thus far, and is 1.4 times the smallest one which is the 86.7-kb cpDNA of *U. linza* (*Uli*) (Wang et al., 2017). To clarify the unique evolutionary trend of *Ulva* plastomes, we built the *Ulva* plastome dataset composed of newly sequenced plastomes and the data deposited in the GenBank database (**Table 1**), with a total of 40 *Ulva* plastomes which represented 19 *Ulva* species from two independent *Ulva* evolutionary lineages (I and II) (Liu and Melton, 2021; Liu et al., 2022a), and compared *Ulva* plastomes with those in other lineages of Ulvophyceae (**Supplementary Table 1**).

**Figure 1:**
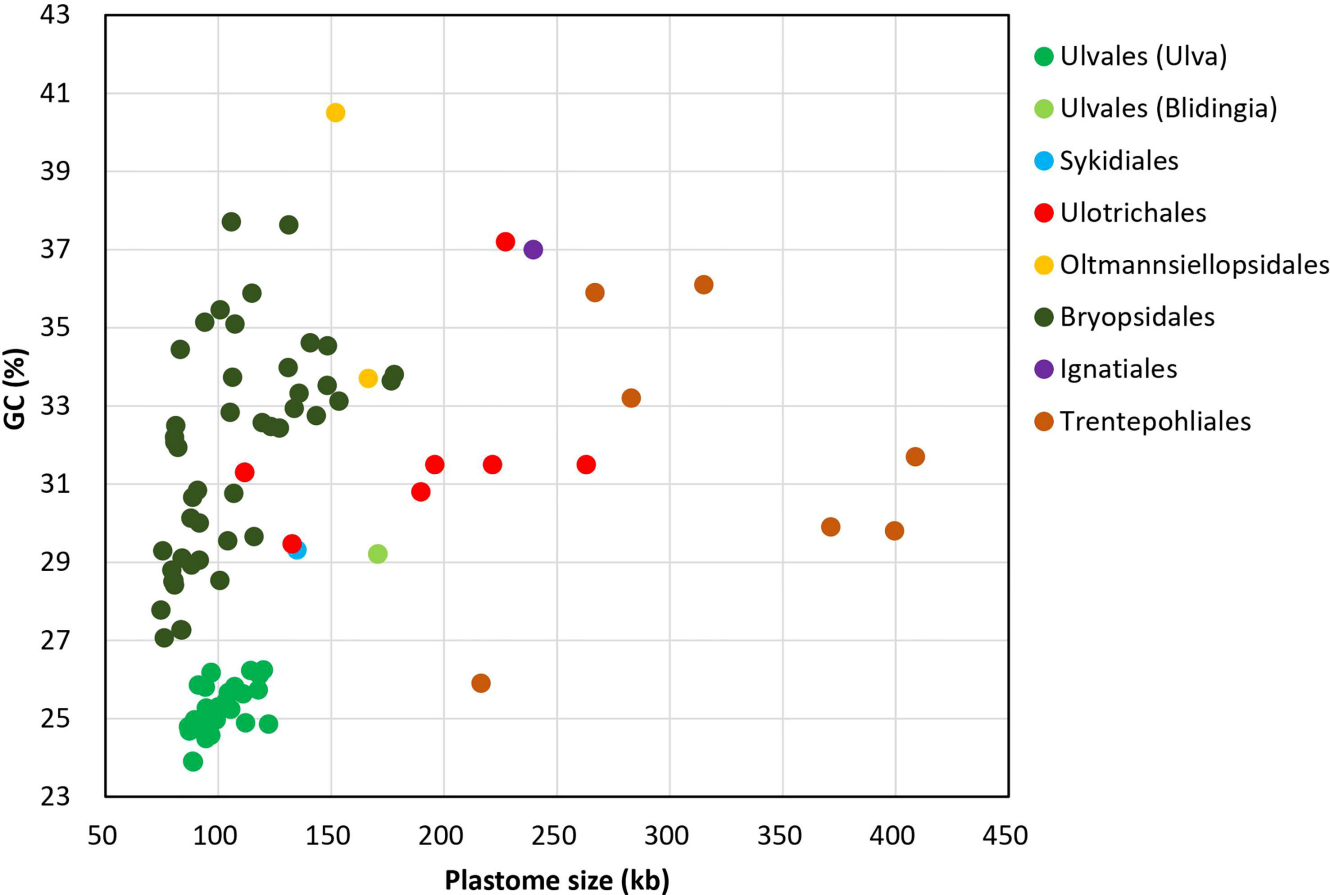
Comparison of GC composition and plastome size in different lineages of Ulvophyceae, including *Ulva* (39 plastomes. *Uco5* was not included here) and *Blidingia* (2) in Ulvales, Sykidiales (1), Ulotrichales (8), Oltmannsiellopsidales (2), Bryopsidales (48), Ignatiales (2) and Trentepohliales (7) (**Supplementary Table 1**).

On the whole, *Ulva* cpDNAs show a clear evolutionary trend of becoming smaller and more compact when compared with all circular complete counterparts in Ulvophyceae (**Figure 1**). The *Ulva* plastomes only encoded a total of 100 conserved canonical genes, including 71 protein-coding genes (PCGs), three rRNA genes and 26 tRNA genes, which are the least among the sequenced circular ulvophycean plastomes. The overall coding regions composed of these 100 canonical genes occupy approximately 71.2 - 72.5 kb in size. Only small repeat sequences were observed to be concentrated either in intergenic spacer regions or intronic regions, and some specific repeat sequences reside in several PCGs (e.g. *rpoB*, *rpoC1*, and *rpoC2*). Unlike those in Sykidiales (*Pseudoneochloris marina*), Ulotrichales, Oltmannsiellopsidales, Ignatiales and Trentepohliales (Pombert et al., 2005; Turmel and Lemieux, 2018; Kim et al., 2019; Fang et al., 2021), most of intergenic regions are relatively short and the rRNA operon-encoding inverted repeat (IR) has been completely lost in *Ulva* plastomes. The minimum size of overall non-coding intergenic regions was only approximately 11.6 kb, which was observed in *U. californica* (*Uca*) (**Supplementary Table 2**).

The ongoing gene loss or transfer can be clearly observed in *Ulva* plastomes. An intact organelle division inhibitor factor gene, *minD* (*orf306*), was observed to be present only in the *U. aragoënsis* (*Uar*) cpDNA (Liu and Melton, 2021). Our further comparative analysis shows that this gene or its residue exists in the *trnL2*-*trnS2* intergenic region of cpDNAs in the *U. aragoënsis*-*U. californica-U.torta* (*Uar*-*Uca*-*Uto*) subclade, while it was completely lost in the other *Ulva* plastomes as well as the *Blidingia* cpDNAs (Gao et al., 2022). The *minD* was split into two parts (*orf68* and *orf209*) in the *Uca* cpDNA, due to a 5-bp insertion mutation, whereas this gene has degenerated more seriously in the *Uto* cpDNAs, leaving only the residue *orf44*. The fracture and degeneration of *minD*, as well as the decreased GC content in its homologous sequences (**Supplementary Table 2**), indicated that there was no selective pressure to retain the integrity of this gene in *Uca* and *Uto*. Considering that homologues of *minD* are present in other lineages of core Ulvophyceae (Turmel and Lemieux, 2018), this gene is likely to have been transferred to the nuclear genome through horizontal transfer in *Ulva* species containing *minD*-lacking plastomes. However, we have not found this gene in the nuclear genome of *U. mutabilis* yet (De Clerck et al., 2018), but it cannot be determined that it does not exist in the nuclear genome, considering the incompleteness of the sequenced *Ulva* genome.

**Figure 2:**
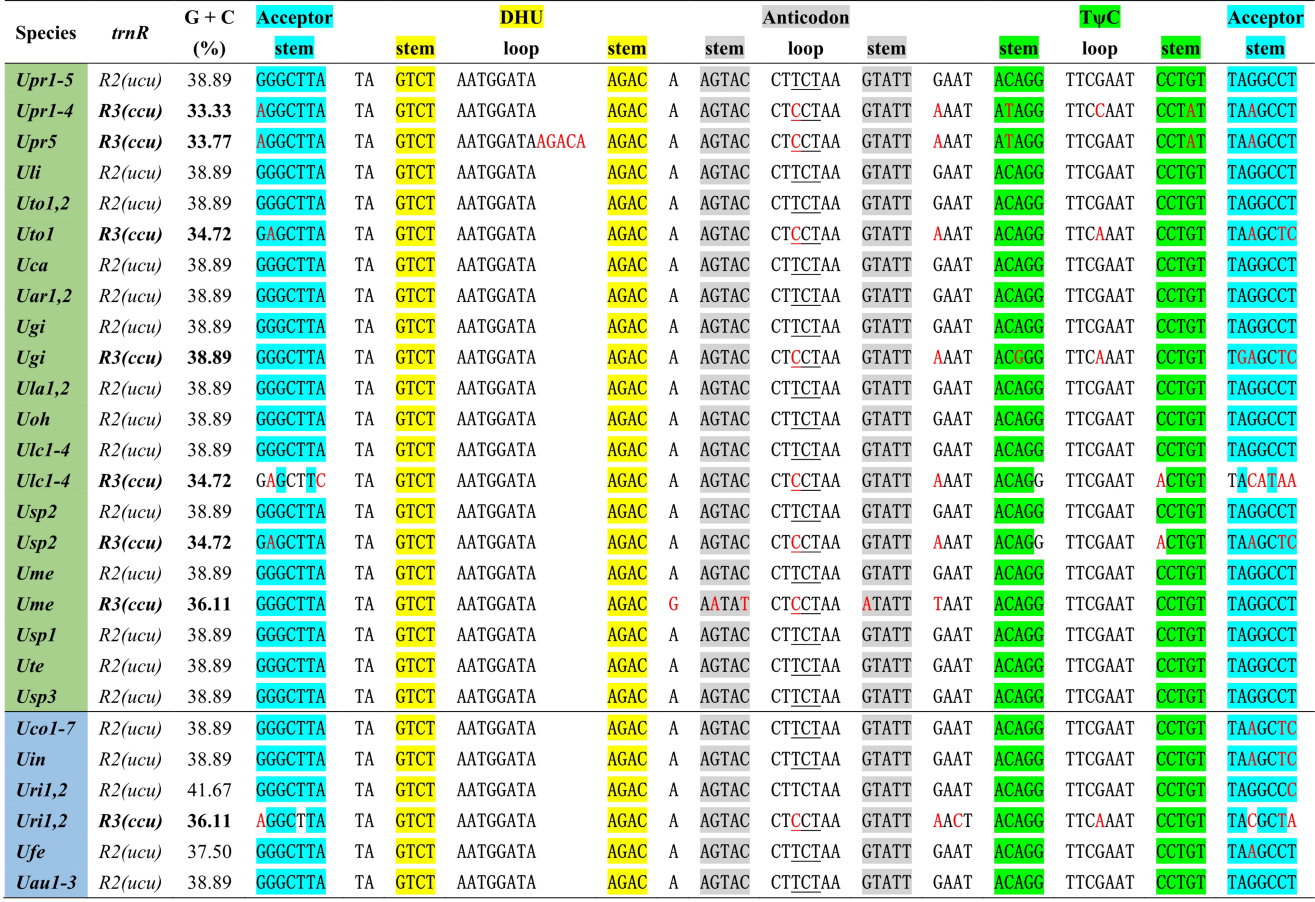
The aligned sequences of *trnR2(ucu)* and *trnR3(ccu)* in *Ulva* plastomes. Shaded nucleotides indicated that bases could be paired. Red letters in the sequence represent base mutations.

**Figure 3:**
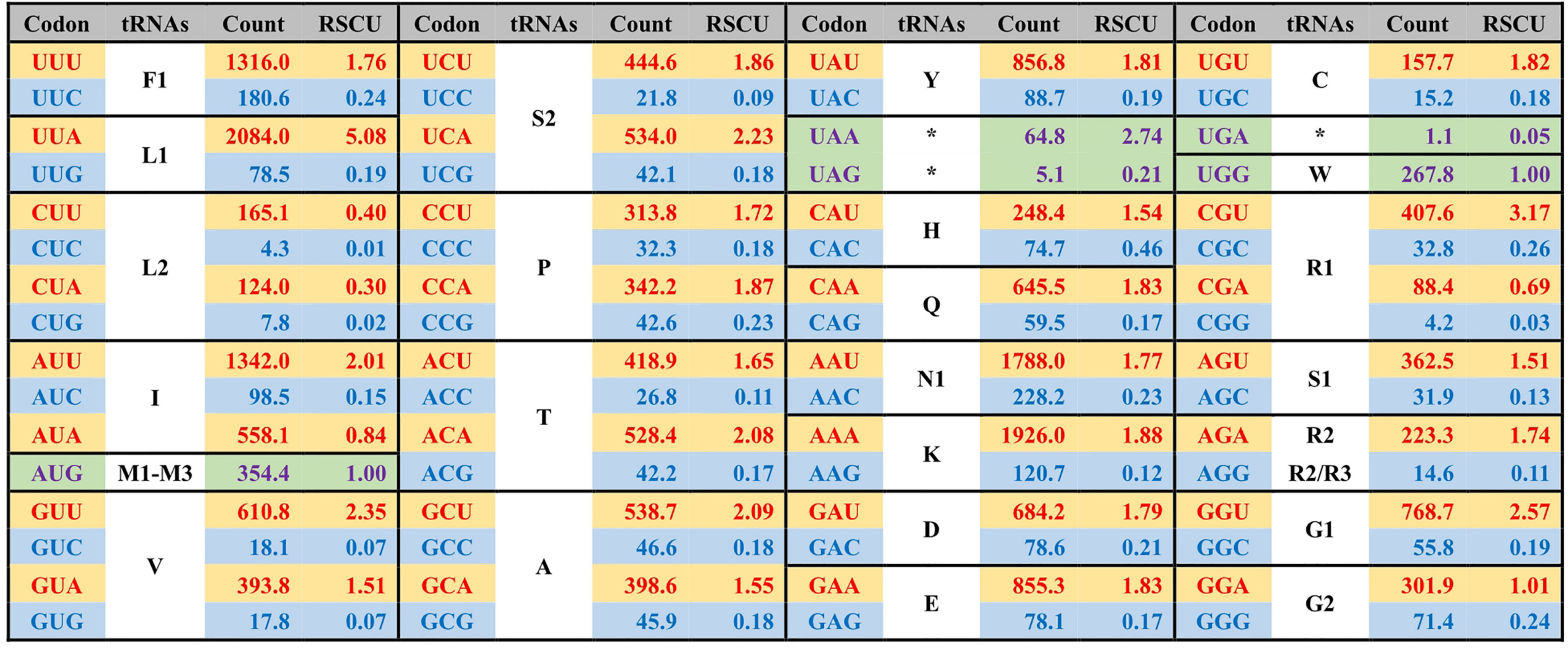
The average codon frequency and relative synonymous codon usage (RSCU) among the 71 core PCGs shared by *Ulva* plastomes. All frequencies are averages over 39 complete *Ulva* cpDNAs, and *Uco5* is not included because some of its PCGs are incomplete (e.g. *rpoA*, *rpoB*, *rpoC1* and *rpoC2*). The serial number of chloroplast tRNA genes is consistent with that previously reported (Liu and Melton, 2021).

One specific *trnR3*(*ccu*) gene is present in plastomes of some *Ulva* species including *U. prolifera* (*Upr1-5*), *U. gigantea* (*Ugi*), *U. rigida* (*Uri1-2*), *U. torta* (*Uto1*), *U. meridionalis* (*Ume*), *U. lacinulata* (*Ulc1-4*) and *Ulva* sp. (*Usp2*) (**Figure 2**). This gene is conservatively located in the downstream region adjacent to *psbC* (Liu and Melton, 2021), but it is situated between *psbA* and *trnT* in the *Uto1* cpDNA. Comparative analysis shows that this gene is highly similar with *trnR2(ucu)*, indicating that it originated from the duplication of *trnR2(ucu)*, and then its anti-codon mutated from UCU to CCU. More mutation sites were detected in *trnR3*(*ccu*) when compared with *trnR2(ucu)*, and the latter maintains a highly conserved sequence in *Ulva* cpDNAs. A 5-bp (ACAAG) duplication mutation was detected in the dihydrouridine (DHU) stem-loop structure of *trnR3*(*ccu*) only in *Upr5*. The rates of sequence evolution for *trnR3*(*ccu*) appear to be dramatically higher than for *trnR2(ucu)*, and the GC contents of *trnR3*(*ccu*) tend to decrease in varying degrees (**Figure 2**), indicating that *trnR3*(*ccu*) is subject to different selection pressures when compared with *trnR2(ucu)*. We found that the 26 core tRNA genes are sufficient to meet all the requirements of protein synthesis in *Ulva* chloroplast genomes (**Figure 3**). Redundant *trnR3*(*ccu*) can be completely replaced by *trnR2(ucu)* in function, which should be the reason why it underwent significantly accelerated sequence evolution or a complete loss that had occurred.

In addition, these IR-lacking *Ulva* plastomes could sporadically incorporate foreign sequences in some specific intergenic regions, and accept group I/II introns in some housekeeping genes, which obviously increased their size. The current size of *Ulva* plastomes was the result of dynamic changes caused by several of the above factors. Especially, marked intraspecific differences in *Ulva* plastome sizes are common, involving gain or loss of introns, integration of foreign fragments and abundance of repetitive sequences (Liu and Melton, 2021).

### Strong selection against GC in *Ulva* plastomes

The GC composition of ulvophycean plastomes varies significantly among different lineages (Turmel et al., 2017; Zhu et al., 2019; Fang et al., 2021), but particularly remarkable is that the GC content of *Ulva* cpDNAs was the lowest among the Ulvophyceae so far, ranging from 23.89 to 26.25% (**Figure 1**), indicating the strong selection against GC to shape the nucleotide composition in *Ulva* plastomes. Due to that GC content showed greatly heterogeneity in distinct regions of *Ulva* plastomes, we analyzed the differences in GC content of overall core-gene coding regions, intronic regions, foreign sequence regions, and non-coding intergenic spacer regions among these 40 *Ulva* cpDNAs.

The GC content is 26.53 - 27.71% in overall coding regions composed of 100 canonical genes (**Supplementary Table 2**). The GC composition of core-gene coding region as well as its total size showed very similar values at intraspecific level or among closely related species, but there are significant differences in the GC content and size of core-gene coding regions among different *Ulva* lineages (**Table 1**; **Supplementary Table 2**). The 26 tRNA regions have the highest GC content (51.32 - 51.88%), followed by the 3 rRNA regions (44.61 - 45.20%) and then the 71 PCG regions (24.50 - 25.82%) (**Supplementary Table 2**).

**Figure 4:**
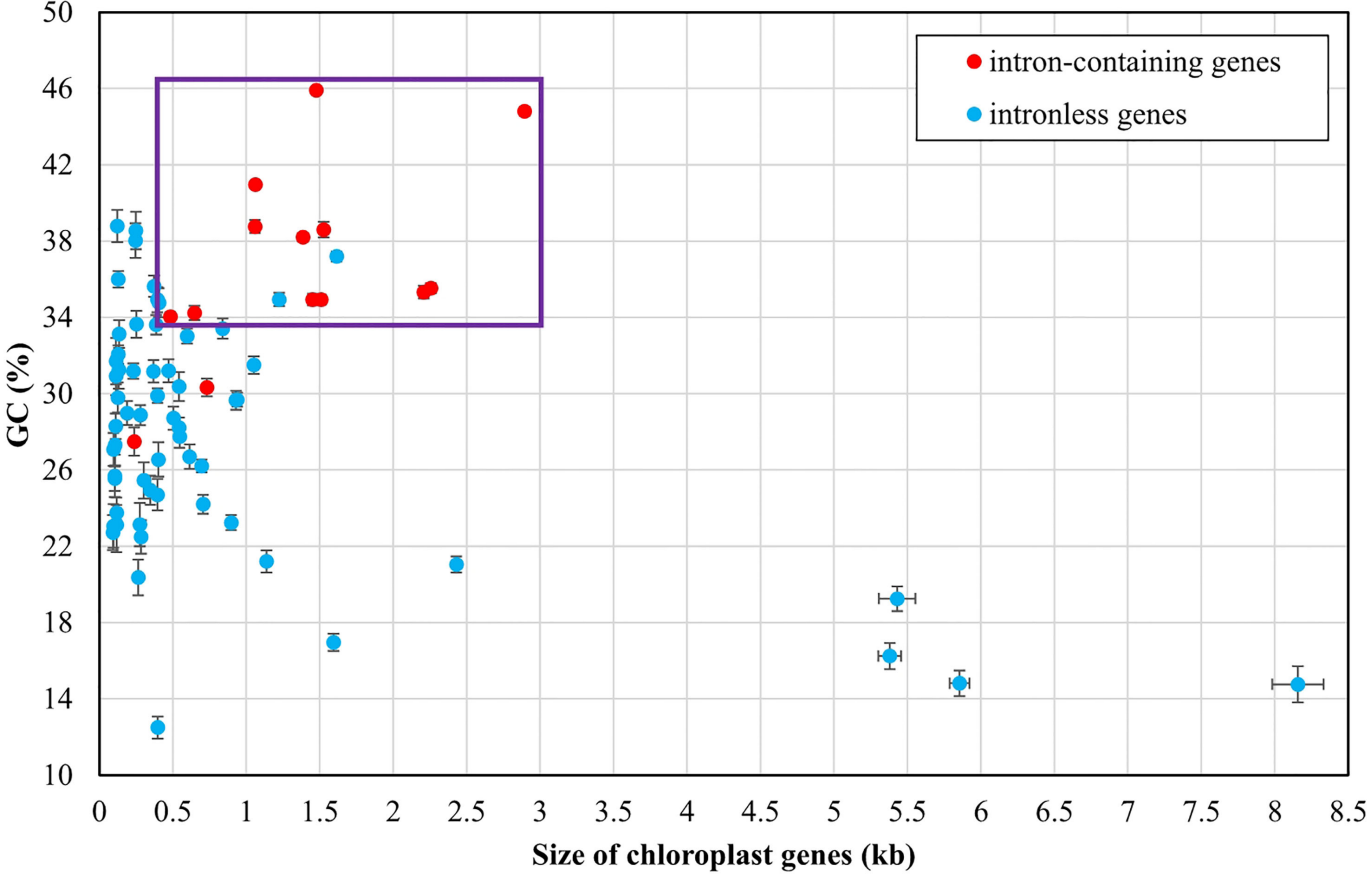
Comparison of GC composition and gene size among 71 PCGs and three rRNAs of *Ulva* plastomes. Error bars represent the standard deviation (SD). Purple box represents the distribution area of most intron-containing genes.

The GC composition is relatively stable in chloroplast rRNAs and tRNAs, but their variations in PCGs fluctuate greatly. Some PCGs related to photosynthesis have much higher GC content, e.g. *psbA* (40.96 ± 0.17%), *psbB* (38.60 ± 0.41%), *psbC* (38.20 ± 0.22%), *psbD* (38.76 ± 0.35%), and *psaC* (38.02 ± 0.91%), while some PCGs with large molecular weight, which are mainly involved in transcription and proteolysis, show very low GC content, e.g. *ftsH* (14.81 ± 0.68%), *rpoA* (16.96 ± 0.45%), *rpoB* (19.25 ± 0.65%), *rpoC1* (16.24 ± 0.69%), and *rpoC2* (14.76 ± 0.95%) (**Figure 4**). On the whole, codon usage pattern in *Ulva* chloroplast PCGs showed a much stronger preference for codons with A or T at the third position (**Figure 3**). The difference in GC content between chloroplast PCGs is mainly determined by their different amino acid composition and the different usage frequency of synonymous codons. The long PCGs with low GC content employ a large number of codons composed of only A and T. For example, the seven most frequently used codons in *rpoC2* are AAU(N), AAA(K), UUA(L), UUU(F), UAU(Y), AUU(I), and AUA(I). However, GC-biased PCGs tend to prefer some codons with C at the third position. For example, UUC(F), AAC(N), AUC(I), UAC(Y) and CAC(H) were used more frequently than their synonymous codons in *psbA*.

The overall intronic regions show great difference in GC composition among different *Ulva* cpDNAs, ranging from 22.59% in *U. linza* (*Uli*) to 34.72% in *U. compressa* (*Uco2*) (**Supplementary Table 2**), as mainly depends on type and content of introns which cpDNA harbors. The GC content of overall foreign sequence regions range from 22.79 to 35.83% in nearly all *Ulva* cpDNAs with the exception of *Usp3* cpDNAs which show much lower values (18.69 - 18.87%) (**Supplementary Table 2**). The GC content in noncoding intergenic regions is obviously the lowest in the range from 8.38% in *U. californica* (*Uca*) to 19.25% in *U. australis* (*Uau1*) (**Supplementary Table 2**).

Low GC content in *Ulva* plastomes is mainly attributed to strong selection pressure driving A + T richness at a genomic level. This selection pressure seems to act on the overall plastome sequences in the microenvironment of *Ulva* chloroplasts, including coding regions, introns, foreign sequences and noncoding regions. The GC composition of all these regions has been markedly reduced when compared with the counterparts in other ulvophycean plastomes (Leliaert and Lopez-Bautista, 2015; Turmel et al., 2016). Plastomes in order Bryopsidales also show a trend of decreasing in size (74.5 - 177.8 kb) and GC content (27.07 - 37.71%) (**Figure 1**), and some species in Bryopsidales adopt the strategy of increasing the number of overlapping regions to make plastomes compact. The lowest GC content in Bryopsidales was 27.07%, which was observed in *Boodleopsis* plastome (MH591104). Its GC content in overall coding region (28.15%) is more than twice that in non-coding region (12.82%), and it was higher than that in coding regions of *Ulva* plastomes (26.53 - 27.71%).

Considering the high energy consumption and high nitrogen demand for GTP and CTP synthesis and the shortening of the sequence length in most PCGs (Mann and Chen, 2010), the advantage conferred by selection against GC observed in *Ulva* plastomes not in other ulvophycean plastomes seems to be more effective in saving the energy cost and serving photosynthesis and biomass synthesis in *Ulva* species, which is more conducive to supporting rapid and abundant growth of *Ulva* species. Meanwhile, the GC content (32.17 - 38.84%) in mitogenomes of *Ulva* species (Liu et al., 2022a; Liu et al., 2022b) does not show a significant difference from those in Ulotrichales and Oltmannsiellopsidales (Turmel et al., 2016). This strong selection of A + T preference seen in plastomes does not appear in nuclear genomes of *Ulva* species (e.g. 57.2% in *U. mutabilis* and 57.3% in *U. compressa*) (De Clerck et al., 2018; Osorio et al., 2022).

### Distribution and diversity of *Ulva* plastome introns

These newly sequenced *Ulva* plastomes harbor different intron contents ranging from two in *U. aragoënsis* (*Uar1*) to 16 in *U. meridionalis* (*Ume*), occupying 3.5 - 17.1% of cpDNAs, which are in the range of the reported *Ulva* plastomes (Liu and Melton, 2021). To further understand the evolutionary trend of *Ulva* chloroplast introns in distribution and diversity, we systematically excavated and compared introns at intrageneric level. A total of 34 intron families were found among these 40 known *Ulva* chloroplast genomes. Among them, 33 intron families were found by RNAweasel (Lang et al., 2007), and only one (intron *rns*-476) was detected by alignment of homologous gene sequences (**Table 2**).

**Table 2:** General features of *Ulva* chloroplast introns detected among the 40 *Ulva* plastomes.

Intron names *	Intron groups	Intron number (*n*)	Intron-encoded proteins (domain)	LAGLIDADG motif	Avg. intron size (SD, bp)	Avg. GC (SD, %)
*atpA*-492	IB (complete)	17	LAGLIDADG	double	1168 (16)	23.24 (0.60)
*atpB*-537	IIB	1	RTM	–	2355	33.38
*atpB*-627	IIB	15	RTM	–	2225 (13)	36.79 (0.93)
*atpB*-696	IIB	17	RTM	–	2372 (12)	36.52 (0.52)
*atpI*-256	IIB	1	RTM	–	2252	36.23
*infA*-62	II (derived)	40 (40) **	–	–	616 (68)	22.69 (1.20)
*petB*-23	IIB	3	RTM	–	2316 (1)	34.66 (0.52)
*petB*-69	IIB	20 (1) **	RTM	–	2207 (92)	35.38 (0.94)
*petB*-169	IIB	2	RTM	–	2459	34.85
*petB*-277	IIB	6	RTM	–	2447 (13)	36.37 (0.41)
*petB*-528	IB (complete)	14 (1) **	LAGLIDADG	double	1265 (14)	23.94 (0.51)
*petD*-87	IIA	4	RTM	–	2428 (11)	36.23 (0.03)
*psaA*-1104	IB (complete)	3	LAGLIDADG	double	1238 (1)	19.43 (0.11)
*psaA*-1605	IB (complete)	2	LAGLIDADG	double	1096	22.45
*psaB*-1050	IB (complete)	15	LAGLIDADG	double	1123 (20)	22.59 (0.45)
*psbA*-179	I (derived, B1)	1	LAGLIDADG	single	695	28.92
*psbA*-750	I (derived, B1)	3 (1) **	T5orf172	–	752 (399)	32.27 (1.22)
*psbB*-489	I (derived, A)	8 (2) **	GIY-YIG	–	880 (250)	24.61 (1.43)
*psbB*-600	IB (complete)	8	LAGLIDADG	double	1301 (41)	24.58 (1.00)
*psbB*-772	I (derived, A)	3 (3) **	–	–	367 (35)	27.25 (2.55)
*psbB*-1022	I (derived, B1)	3	HNH	–	959 (9)	25.36 (0.69)
*psbB-*1352	I (derived, B1)	2	HNH	–	947 (10)	24.19 (1.44)
*psbC*-496	IIB	1	RTM	–	2441	36.71
*psbC*-708	IA	4	LAGLIDADG	single	986 (5)	26.11 (1.22)
*psbC*-882	I (derived, A)	4	GIY-YIG	–	926 (4)	24.72 (0.52)
*psbD*-740	I (derived, A)	11	GIY-YIG	–	1039 (21)	24.64 (0.77)
*psbD*-1034	I (derived, A)	2	GIY-YIG	–	1006 (108)	24.28 (1.13)
*rnl-*1893a	IB (complete)	19	LAGLIDADG	single	765 (3)	30.38 (1.01)
*rnl-*1893b	IB (complete)	3	LAGLIDADG	double	1007 (5)	25.03 (0.40)
*rnl*-2225	IB (complete)	22	LAGLIDADG	single	966 (24)	26.79 (0.26)
*rnl*-2463	IB (complete)	4	LAGLIDADG	single	1013 (1)	26.40 (0.22)
*rnl*-2556	I (derived, B2)	5	LAGLIDADG	single	757 (24)	27.66 (0.43)
*rns*-476	I (unknown)	4	LAGLIDADG	single	1007 (3)	24.39 (0.19)
*rns*-499	IA3	5 (3) **	LAGLIDADG	double	938 (779)	27.63 (4.10)

* Intron names were defined as host gene plus insertion site which was determined by comparing homologous genes relative to the plastome of *U. compressa* (MW353781) (Liu and Melton, 2021).

** Numbers in parentheses indicate the number of introns with severe IEP degradation or loss.

The *Ulva* chloroplast introns were detected at 33 insertion sites of 14 host genes including *atpA* (1 site), *atpB* (3), *atpI* (1), *infA* (1), *petB* (5), *petD* (1), *psaA* (2), *psaB* (1), *psbA* (2), *psbB* (5), *psbC* (3), *psbD* (2), *rnl* (4) and *rns* (2) (**Table 2**). Obviously, intron densities varied widely among chloroplast genes at interspecific and intraspecific levels (**Supplementary Figure 1**). These introns were mainly distributed in two rRNA genes (*rnl*, and *rns*) and some more conserved PCGs involving photosystem I and II, electron transport and ATP synthesis, indicating that different functional groups of genes have different propensities for intron insertion. Further comparative analysis showed that introns preferentially resided in conserved housekeeping genes with high GC content (usually more than 34%) and long size (usually more than 0.5 kb) (**Figure 4**). We speculate that this may be related to higher GC content in target site sequences required for IEP recognition in host genes and more target sites contained by long GC-rich genes. However, some PCGs (e.g. *rbcL*, and *tufA*) with the above similar characteristics have high expression in chloroplasts and tend to resist intron invasion to economically and effectively ensure unnecessary consumption and time cost in transcription and processing (Jeffares et al., 2006).

**Figure 5:**
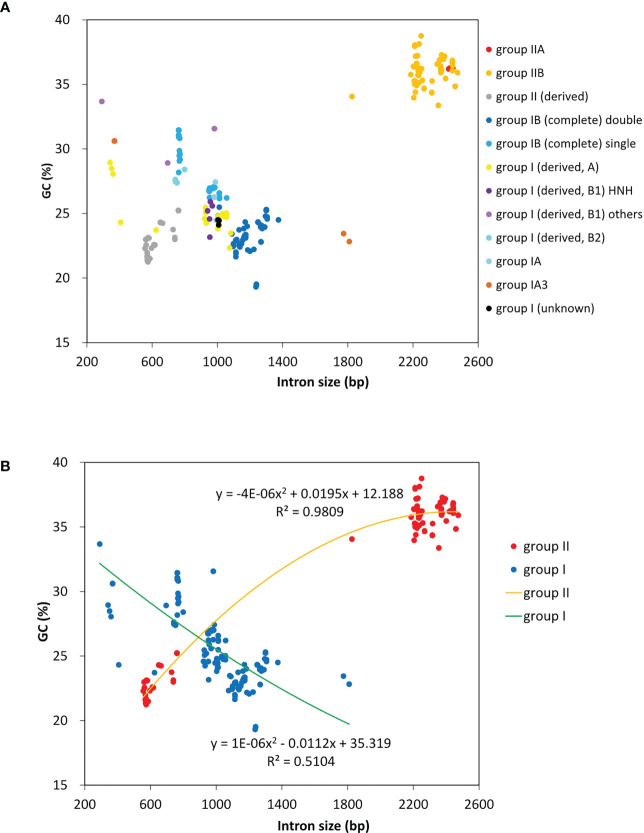
The GC composition and size of introns detected among 40 *Ulva* plastomes. **(A)** Comparison of GC content and size among different types of introns. **(B)** Comparison of GC content and size between group I and group II introns.

A total of 23 intron families were observed to belong to group I introns, and the remaining 11 were group II introns. Eight intron families including intron *atpB*-537, *psaA*-1605, *psbA*-179, *psbB*-1022, *psbB-*1352, *psbC*-708, *psbD*-1034 and *rnl-*1893b were found for the first time in Chlorophyta. The size and GC content of different intron families fluctuate markedly in *Ulva* plastomes (**Table 2**), which are largely determined by type of introns and degeneration degree of intron-encoded proteins (IEPs) (**Figure 5A**). Except for the degenerated group II (derived) intron *infA*-62 (Liu and Melton, 2021), the size of all other group II introns is significantly longer than that of group I introns, due to the different size of their IEP genes (**Table 2**). The GC content is positively correlated with group II intron size with the excellent coefficient of determination (R^2^ = 0.9809) (**Figure 5B**). Group IIA/IIB introns have the high GC content ranging from 33.38% in intron *atp*-537 to 36.79 ± 0.93% in intron *atpB*-627, while the GC content of group II (derived) intron *infA*-62 is only 22.69 ± 1.20% mainly due to the loss of the entire IEP (**Table 2**). Contrary to the group II introns, there is a weak negative correlation between GC content and size in group I intron (**Figure 5B**). The GC content in group I introns shows complex changes, which is mainly due to the diversity of types (related to secondary structure) and IEPs.

Group IIA/IIB introns harbor a reverse transcriptase/maturase (RTM) gene in *Ulva* plastomes. The vast majority (93.8%) of group I introns encode an intact IEP which is the member of the LAGLIDADG or GIY-YIG or HNH homing endonuclease (LHE or GHE or NHE) families. However, the IEPs from intron *psb*A-750 do not exhibit significant sequence similarity to common homing endonuclease families (e.g. LHE, GHE, and NHE), but contain a conserved T5orf172 domain which occurs in a stand-alone protein form in phage, virus and bacteria and is also found in DNA-binding regulatory proteins of bacterial and eukaryotic DNA viruses (Iyer et al., 2002). All chloroplast GHEs were encoded by group I (derived, A) introns, while the IEPs encoded by group I (derived, B1) introns showed diverse protein types including NHE, LHE, and T5orf172 domain-containing homing endonuclease (THE). All of chloroplast group IB introns and other group I introns encoded an LHE with one or two LAGLIDADG motifs.

Almost all introns displayed sporadic distribution pattern in *Ulva* plastomes, due to their nature of homing and mobility. Only the chloroplast intron *infA*-62 is an exception. This intron is shared by all *Ulva* plastomes, but absent in *Blidingia* cpDNAs, indicating that it might be acquired after its divergence from *Blidingia*. This intron has completely lost the ability to move and has been trapped in *infA*, because of its severe degeneration and the loss of IEP. This intron co-evolved with *infA* and showed a faster evolution rate than the host gene. Introns from the same insertion site were previously observed to be homologous among organelle genomes in *Ulva* (Liu and Melton, 2021; Liu et al., 2022a). However, two different intron families, intron *rnl-*1893a and *rnl-*1893b, were found to be present in the same insertion site. Although both of them belong to group IB intron and share similar secondary structure of ribozyme components, but their primary sequences and IEPs are markedly different. The IEPs in intron *rnl-*1893a were LHEs with only one LAGLIDADG motif, while those in intron *rnl-*1893b contained two LAGLIDADG motifs (**Table 2**). These facts indicated that these two different LHEs should recognize the same target site in *rnl*, although they can be a homodimer and a monomer (Haugen et al., 2005), respectively.

### Novel insights into integration of foreign sequences and rearrangement of *Ulva* plastomes

Comparison of plastome intergenic regions shows that *Ulva* cpDNAs experienced frequent insertion of foreign DNA sequences which usually harbor some specific open reading frames (*orf*s), as were important indicators for insight into their source. The largest *U. meridionalis* (*Ume*) cpDNA contained 14.6-kb foreign sequence which encoded 15 specific *orf*s (**Supplementary Figure 2**), accounting for 11.9% of plastome. To elucidate the origin of exogenous sequences and their relationships, we systematically compared the sequence characteristics of large intergenic regions and the distribution of free-standing *orf*s among these 40 *Ulva* plastomes. A total of 154 specific free-standing *orf*s as well as many homologous residue DNA sequences of some specific *orf*s, which have no similarity to chloroplast canonical genes, were detected in intergenic regions of these 40 *Ulva* cpDNAs. These specific *orf*s were not randomly distributed but mainly located in some specific intergenic regions (e.g. *psbA*-*psbB*, *trnT*-*psbA*, *psbB*-*psbD*, *psbC*-*psbB*, *trnS2*-*psbC*, *trnM3*-*psbD*, *psbC*-*trnM3*, *trnL2*-*psbD*, *trnL2*-*trnM3*, *trnM1*-*trnE*, and *trnW*-*psaJ*) (Liu and Melton, 2021), indicating that these intergenic regions should be hot spots for the invasion of foreign sequences.

**Figure 6:**
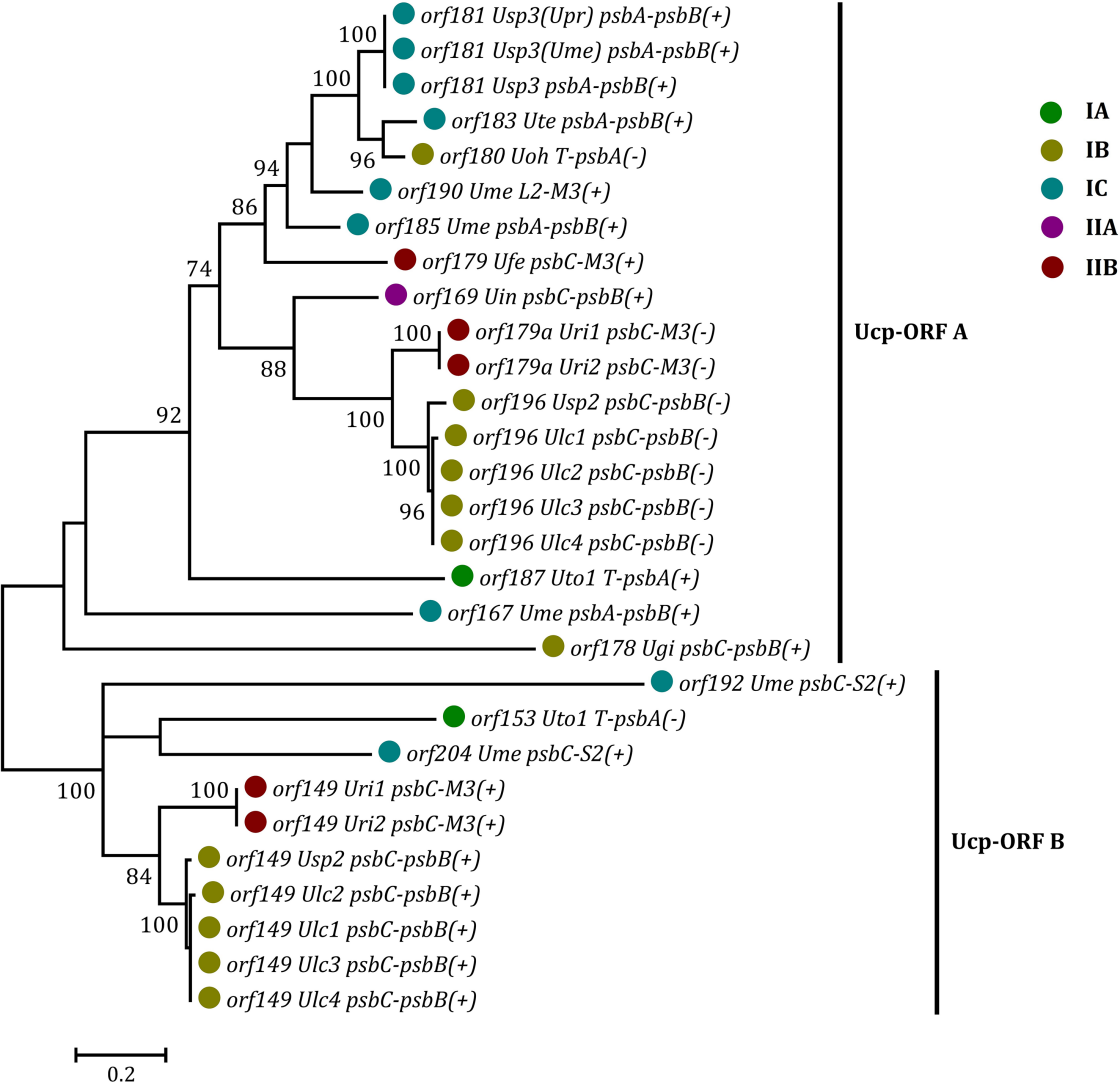
Phylogenetic analysis of the 29 full-length free-standing Ucp-ORFs found in *Ulva* plastomes. The bootstrap support values greater than 70% were displayed at branches. Branch lengths were proportional to the amount of sequence change, which were indicated by the scale bar below the trees. Different colored circles represent different *Ulva* lineages.

**Figure 7:**
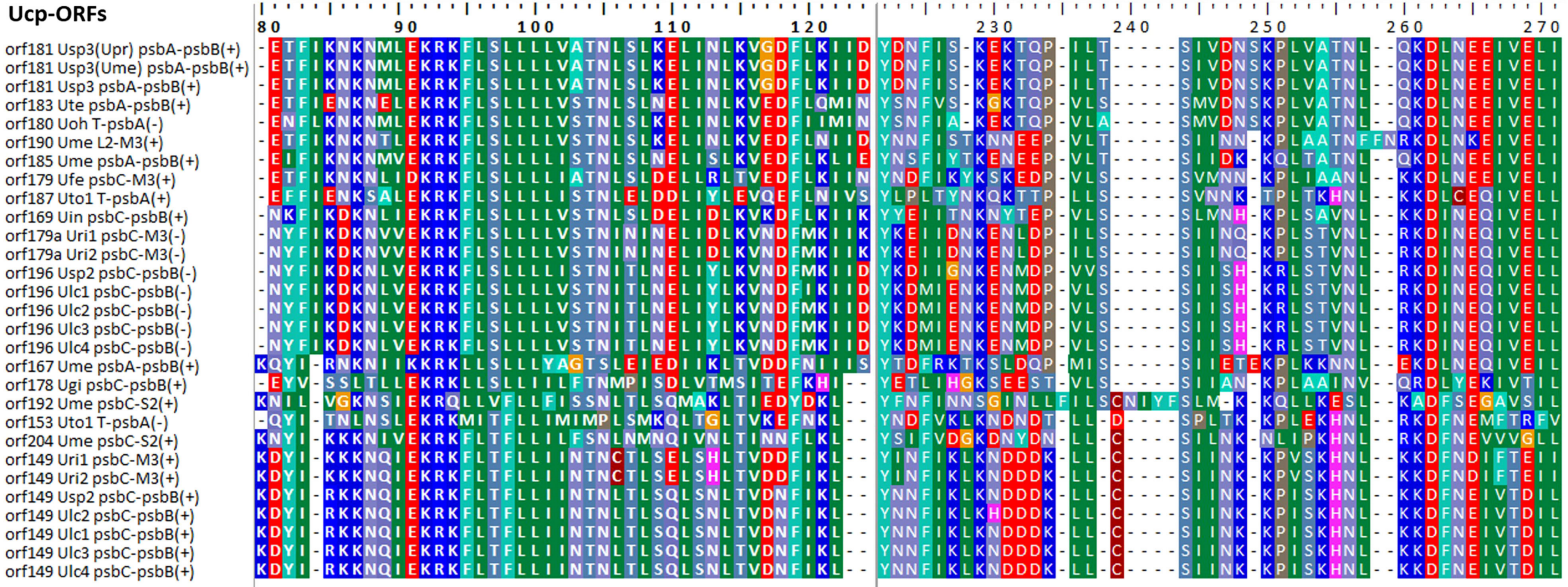
The conserved domain shared by the 29 Ucp-ORFs detected in *Ulva* plastomes.

Some foreign DNA sequences integrated into different intergenic regions of *Ulva* plastomes harbor homologous *orf*s with significant high similarity (**Supplementary Table 3**). These facts indicated that these foreign sequences could have been derived from the same origin. It is very similar to the finding in *Ulva* mitogenomes where the derived foreign sequences mainly originated from mitochondrial plasmid DNA (Liu et al., 2022a; Liu et al., 2022b). Among these specific chloroplast homologous *orf*s, three classes of *orf*s show high similarities to the full length or partial sequences of putative bacterial tyrosine-type recombinase/integrase (*tri*), NAD-dependent DNA ligase (*lig*), and phage/plasmid DNA primase (**Supplementary Table 3**), respectively, based on tblastn search, which were also detected in cpDNAs of some siphonous green algae (Bryopsidales) (Leliaert and Lopez-Bautista, 2015; Cremen et al., 2018). It is worth noting that a new class of specific free-standing *orf*s was found only in *Ulva* plastomes, which we named Ucp-*orf*. A total of 29 full-length Ucp-*orf*s were detected in 17 of 40 *Ulva* plastomes, which belonged to different *Ulva* lineages (**Figure 6**), and none of such *orf* was found in other ulvophyceaen cpDNAs sequenced thus far. Interestingly, all of Ucp-*orf*s reside in several intergenic regions where genome rearrangement occurred. In the *Ume* cpDNA, there are five Ucp-*orf*s in three intergenic regions (i.e. *psbA*-*psbB*, *psbC*-*trnS2*, and *trnL2*-*trnM3*). One conserved domain was shared by all of these Ucp-*orf*s (**Figure 7**), but no information on its function can be obtained based on blastp search. In addition, the remaining *orf*s contain some recognizable protein domains acting on DNA or RNA, but their functions are still unknown (**Supplementary Table 3**).

**Figure 8:**
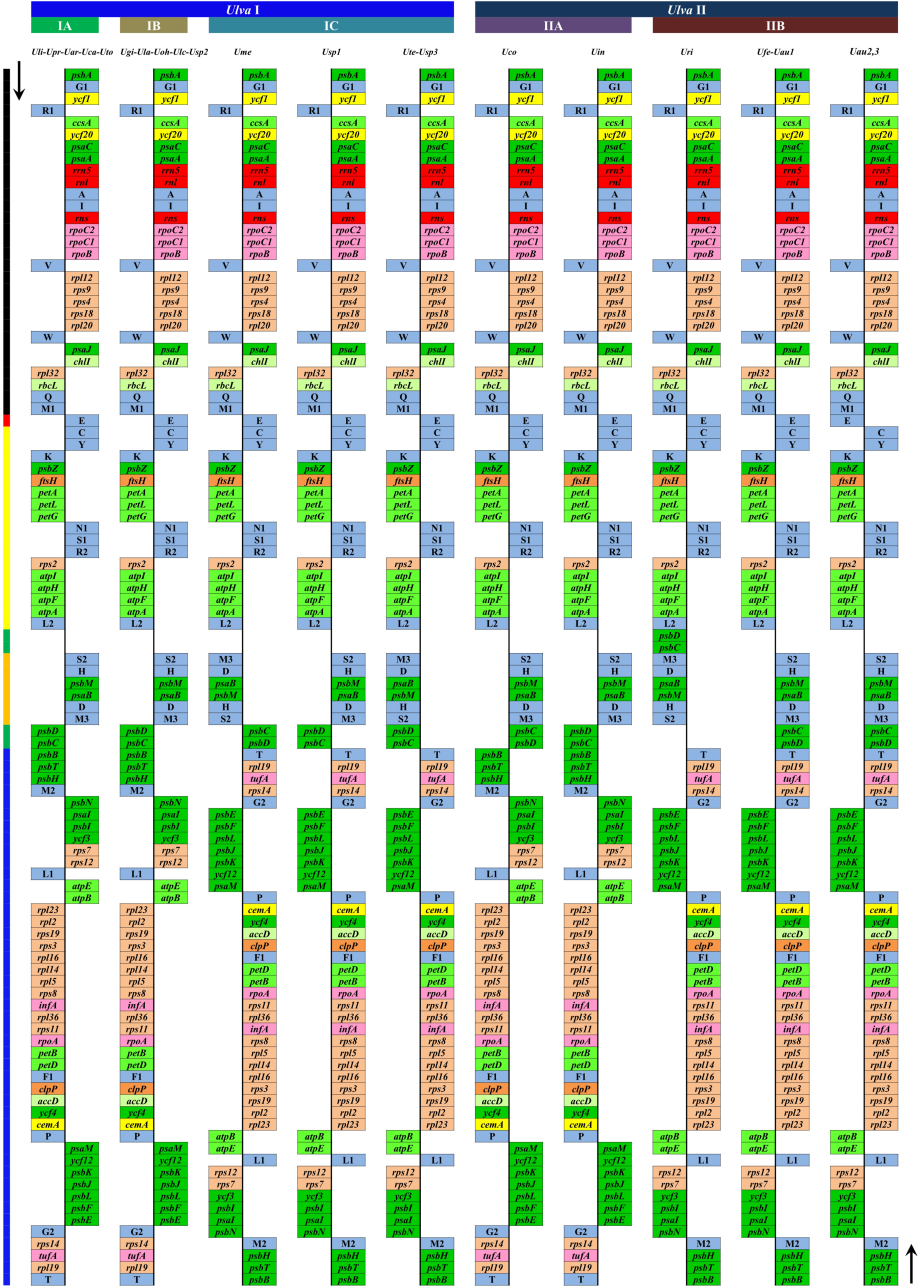
Comparison of plastome organization and gene order between *Ulva* lineage I and II. Thick lines with different colors on the left represented different gene blocks. Thick lines with different colors on the top represented different *Ulva* lineages. The arrows indicated the direction of gene transcription. Only canonical genes were shown as filled boxes in different colors representing different gene types.

The plastome architecture is not as conserved as that of mitogenomes in *Ulva* species (**Supplementary Figure 2**), but has experienced several rearrangement events to varying degrees (Liu and Melton, 2021). It is worth noting that the intergenic regions where foreign sequences frequently invade match well with the regions where plastome rearrangement occurs. Six conserved gene blocks can be detected in *Ulva* cpDNAs by comparing the plastome structure, and chloroplast genome recombination frequently occurs in the regions on both sides of the *psbD*-*psbC* gene block, the upstream region of *psbB* and the downstream region of *trnT* (**Figure 8**). These regions are exactly the regions where foreign DNA sequences are inserted most frequently. It seems that the invasion of foreign fragments causes the instability of genome architecture and triggered inversion of some gene blocks in *Ulva* cpDNAs. The *Ulva* chloroplast genomes belong to IR-lacking cpDNAs, and the IR was supposed to play an important role in stabilizing the architecture of cpDNAs (Turmel and Lemieux, 2018). The invasion of foreign sequences, especially in the context of IR loss, seems to be an important driving force for *Ulva* genome rearrangement.

These acquired *orf*s display a different evolutionary trend from the chloroplast canonical genes. The levels of sequence divergence among homologous specific *orf*s or foreign DNA sequences in *Ulva* cpDNAs greatly exceed those observed in chloroplast genes. Frequent insertion and deletion mutations lead to serious fracture and degeneration of these sequences, accompanied by reduced GC content (**Supplementary Table 2, 3**). These evidences show that their existence is not a necessary requirement of *Ulva* chloroplast genomes, and there is no selection pressure to maintain their existence. Differential GC content can be used as an indicator to distinguish the background genome and the non-self (or introduced) DNA sequence (Mann and Chen, 2010). Because of rapid evolution and high divergence of derived foreign sequences, their GC content decreased at varying levels, as depends on their evolution time and rate after their insertion into different *Ulva* plastomes. Their changes from heterogeneity to homogeneity caused by rapid evolution make it difficult to distinguish some foreign sequences that completely lose coding ability from non-coding intergenic regions. The fate of these integrated foreign sequences will most likely be accelerated evolution and eventually lose, which reminds us of the similar phenomena observed in the *Ulva* mitogenomes where the frequently inserted plasmid-derived sequences underwent multiple mutations and rapid degeneration (Liu et al., 2022a; Liu et al., 2022b).

### Novel insights into plastome architecture and phylogenomic analysis

**Figure 9:**
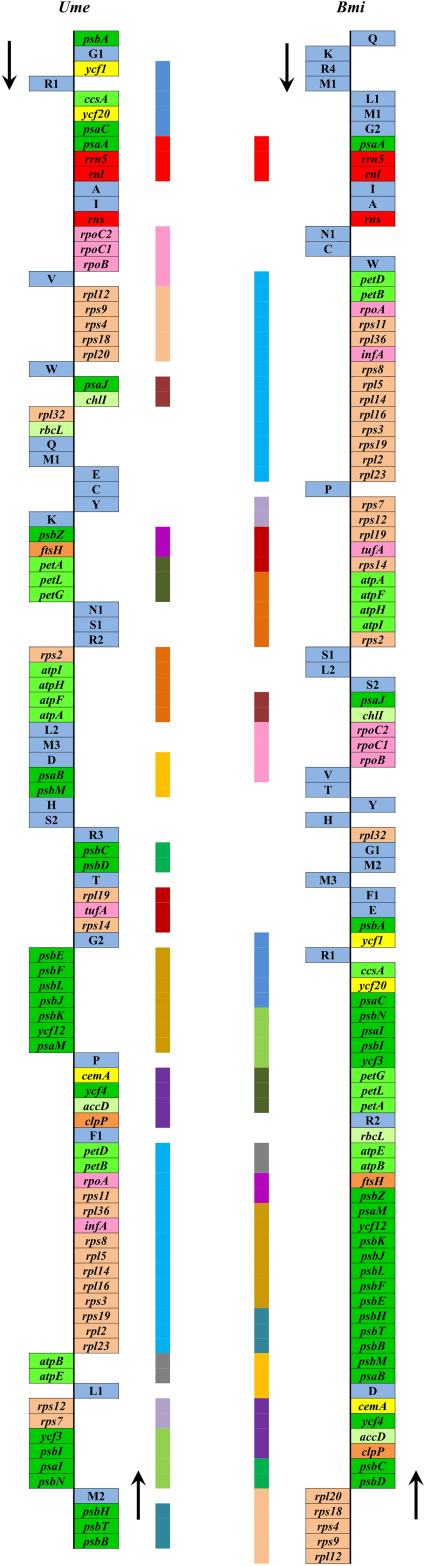
Comparison of plastome organization and gene order between *Ulva meridionalis* (OP985133) and *Blidingia minima* (MK408749 and MT948112). Thick lines with different colors between two plastomes represented different gene blocks. The arrows indicated the direction of gene transcription. Only canonical genes were shown as filled boxes in different colors representing different gene types.

Due to the limited data at present, it is difficult to reconstruct the plastome structure of the common ancestor of *Ulva* species. Based on comparative analysis of architectures in sequenced Ulvales-Sykidiales-Ulotrichales cpDNAs to date, plastomes have completely lost the IR in Ulvales (e.g. *Ulva* species and *B. minima*) (**Figures 8**, **9**), which is a remarkable difference from those in Sykidiales (e.g. *P. marina*) and Ulotrichales carrying identical or non-identical IR copies (Turmel et al., 2017; Kim et al., 2019). Gene order and gene distribution show some new characteristics in these IR-lacking plastomes. In *Ulva* plastomes, gene clusters show a staggered distribution pattern on two strands (**Figure 8**), while in *B. minima* plastomes (MT948112 and MK408749), gene clusters tend to accumulate on one main strand and only one gene cluster (*rpl20-rps8-rps4-rps9-rpl12*) and 13 tRNAs are transcribed on another strand (**Figure 9**). We found that gene partitioning pattern changed after the loss of IR and distribution range of gene clusters became larger, indicating that genome rearrangement was more extensive and more frequent in IR-lacking Ulvales plastomes. Many new gene clusters, e.g. *rpl23-rpl2-rps19-rps3-rpl16-rpl14-rpl5-rps8-infA-rpl36-rps11-rpoA-petB-petD*, *psaC-ycf20-ccsA-trnR1-ycf1-psbA*, *rns-trnI-trnA-rnl-rrn5-psaA*, *trnV-rpoB-rpoC1-rpoC2*, *ycf3-psbI-psaI-psbN*, and *psbB-psbT-psbH*, were observed to be shared only by plastomes of *Ulva* and *Blidingia* (**Figure 9**), but they did not appear in plastomes of *P. marina* and other ulvophycean species (e.g. Pombert et al., 2005; Pombert et al., 2006; Leliaert and Lopez-Bautista, 2015; Turmel et al., 2017; Fang et al., 2021), indicating they have been formed and maintained in their common ancestor after divergence from *P. marina*. These findings provide important clues for us to understand genome structure and gene order of early IR-lacking plastomes in Ulvales.

**Figure 10:**
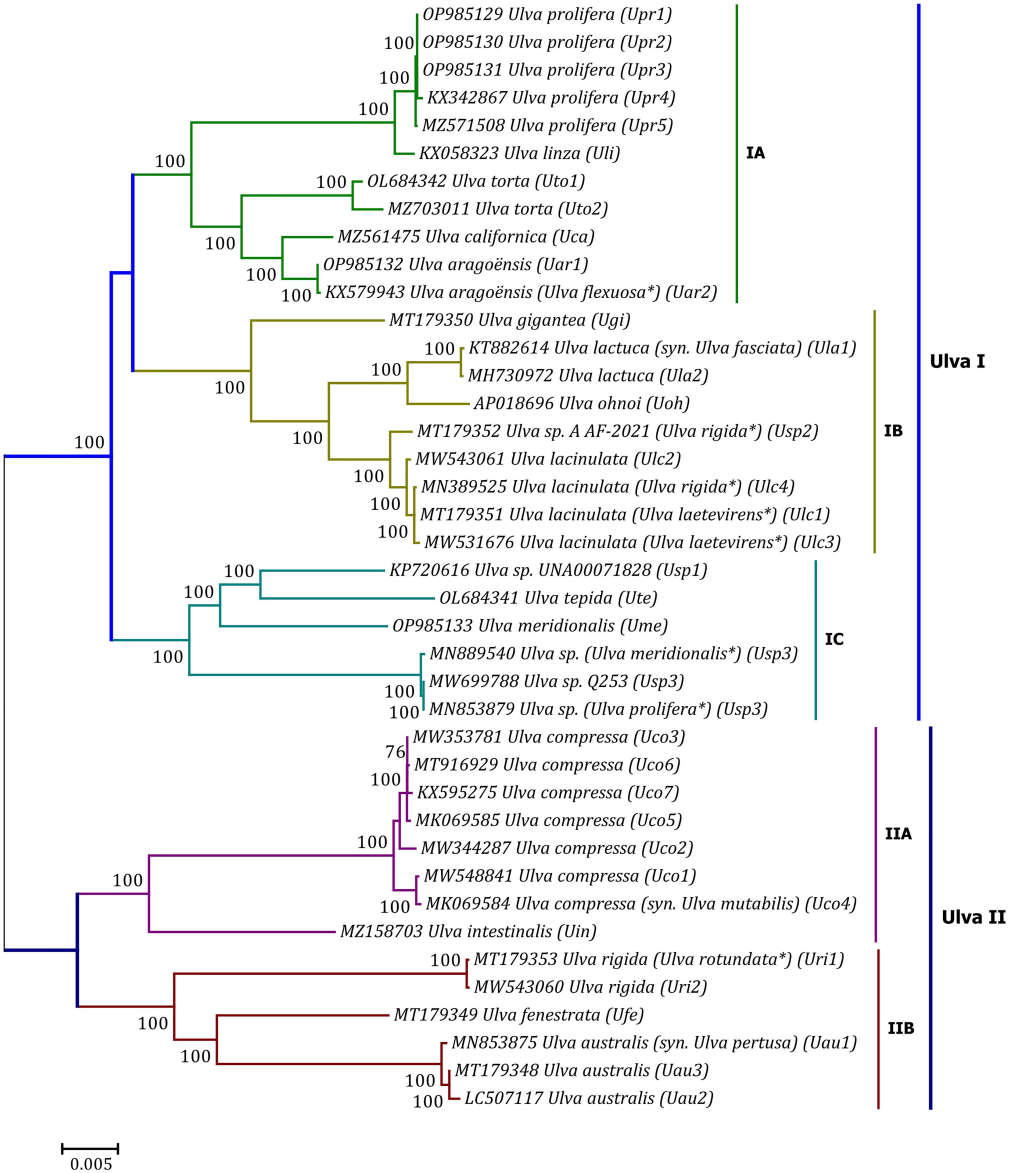
Unrooted phylogenomic tree based on Maximum Likelihood (ML) analysis of the nucleotide (nt) sequences of the 100 common genes in the 40 *Ulva* plastomes. The bootstrap support values greater than 70% were displayed at branches. Branch lengths are proportional to the amount of sequence change, which are indicated by the scale bar below the trees. The asterisk indicates that the species name has been corrected.

**Figure 11:**
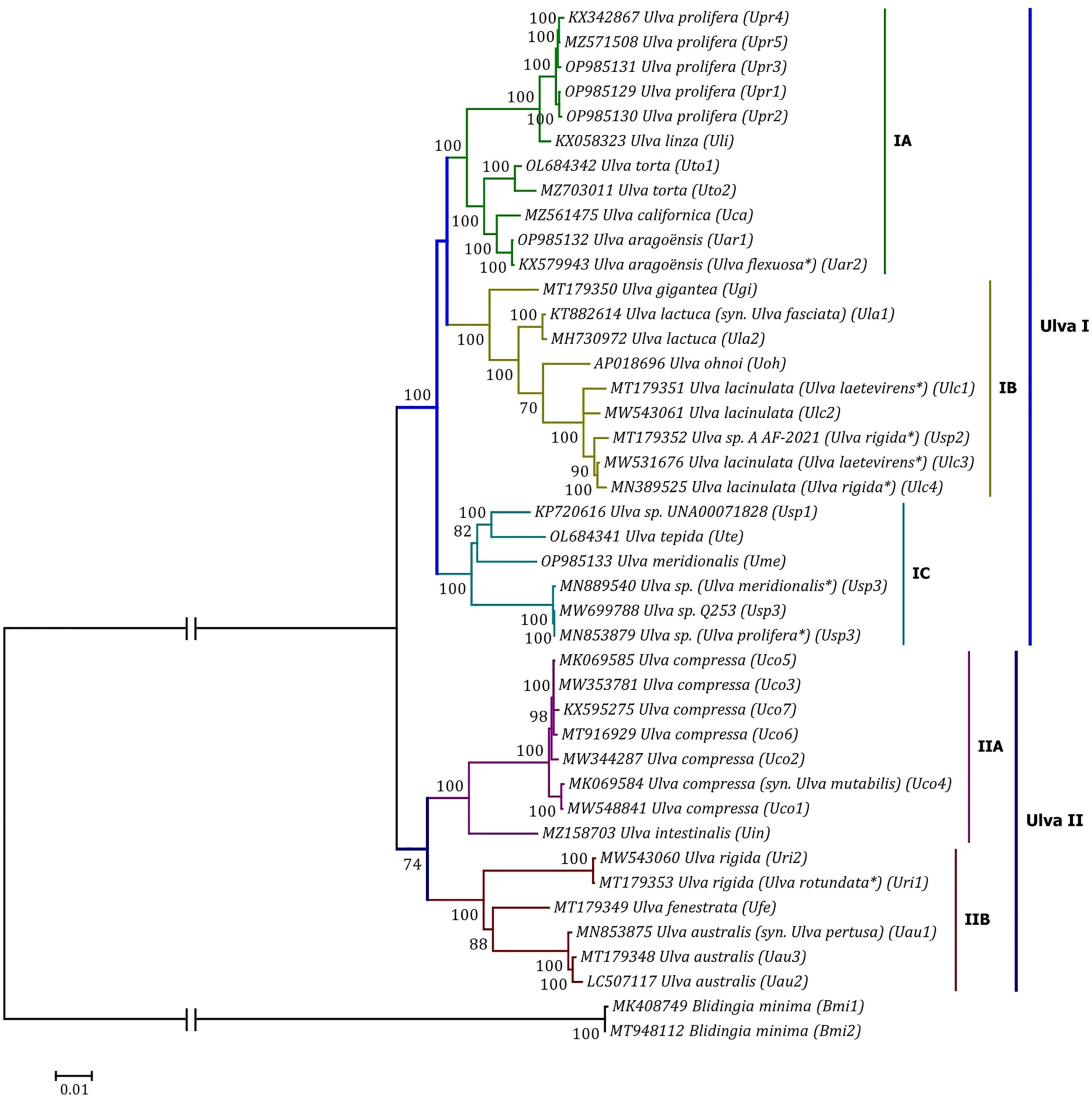
Phylogenomic tree based on Maximum Likelihood (ML) analysis of the amino acid (aa) sequences of the 71 common PCGs in the 40 *Ulva* plastomes. The bootstrap support values greater than 70% were displayed at branches. Branch lengths are proportional to the amount of sequence change, which are indicated by the scale bar below the trees. The tree was rooted with *Blidingia minima* as the outgroup. The asterisk indicates that the species name has been corrected.

Phylogenomic analyses of two *Ulva* plastome datasets (nt sequences of 100 canonical genes and aa sequences of 71 PCGs) showed that the common ancestor of *Ulva* species had a very early internal divergence and split into two major evolutionary lineages (*Ulva* I and II) (Liu and Melton, 2021). *Ulva* lineage I has evolved into at least three independent clades (IA, IB and IC), and *Ulva* lineage II into at least two clades (IIA and IIB) (**Figures 10**, **11**). The inversion of *psbD-psbC* gene cluster is the most significant difference between lineage I and II in gene order, but the continuous inversion of this gene cluster makes the gene order in the *Ulva intestinalis* (*Uin*) plastome completely consistent with those in IA and IB clades (**Figure 8**). *Ulva* IA clade contains the *U. linza-prolifera* (LP) complex which harbor the *rps19* gene with GTG start code and the *Uar*-*Uca*-*Uto* subclade which is the only *minD*-containing *Ulva* lineage. Plastomes in IA and IB clades shared the identical gene order, but were different from those in IC clade. In the IC plastomes, two gene clusters composed of 45 genes from *psbB* to *trnT(ugu)* and six genes (*trnM3-trnD-psaB-psbM-trnH-trnS2*) were inverted respectively, and then the latter had a secondary inversion in *Usp1* and the *psbD-psbC* gene block was inverted in *Ume*. Like plastomes in the IC clade, the large gene cluster containing 45 genes was also inverted in the IIB clade but not in IIA (**Figure 8**; **Supplementary Figure 3**). Because of frequent inversion of gene clusters in plastomes, the gene order cannot well reflect their evolutionary relationship between *Ulva* species (Wang et al., 2021).

Our results of phylogenomic analysis well supported taxonomic revisions of some species names at the genomic level (**Figures 10**, **11**), e.g. *U. mutabilis* Föyn, *U. pertusa* Kjellman and *U. fasciata* Delile are taxonomic synonyms of *U. compressa* Linnaeus (Steinhagen et al., 2019), *U. australis* Areschoug (Couceiro et al., 2011) and U. lactuca Linnaeus (Hughey et al., 2019), respectively. It is worth emphasizing that our results show that eight of 40 *Ulva* plastomes were assigned wrong species names reported at first (Liu J. et al., 2020; Wang et al., 2020; Fort et al., 2021; Hughey et al., 2021). The fact that inaccurate species identification occurred frequently leads to incorrect *Ulva* species names matched by DNA sequences deposited in the GenBank database. Therefore, the way to fundamentally eliminate the mismatch between species names and sequences is to use molecular marker technology, especially the comparative analysis of organelle genomes, to more clearly reveal the genotype differences between individuals of *Ulva* species, especially the closely related species.

## Conclusion

The accumulation of *Ulva* cpDNA data provides us with an opportunity to decipher the unique evolution of plastomes in these globally distributed green macroalgae. In this study, more new insights into plastome evolution of *Ulva* species have been gained. First, *Ulva* plastome evolution reflects the strong selection pressure driving the compactness of genome organization and the decrease of overall genomic GC content. The overall plastome sequences including canonical genes, introns, derived foreign sequences and non-coding regions show a synergetic decrease in GC content at the varying degree. Fast degeneration of plastome sequences including non-core genes (*minD* and *trnR3*), derived foreign sequences, and noncoding spacer regions was accompanied by the marked decrease of their GC composition. Second, introns preferentially resided in conserved housekeeping genes with high GC content and long length in *Ulva* plastomes. It might be related to high GC content in target site sequences required for IEP recognition and more target sites contained by long GC-rich genes. Third, many foreign DNA sequences integrated into different intergenic regions harbor some homologous specific *orf*s with high similarity, indicating that they could have been derived from the same origin. Fourth, the invasion of foreign sequences seems to be an important driving force for plastome rearrangement in these IR-lacking *Ulva* cpDNAs. It seems that the invasion of foreign fragments causes the instability of genome architecture and triggered inversion of some gene blocks in *Ulva* cpDNAs. Finally, gene partitioning pattern changed after the loss of IR, and genome rearrangement was more extensive and more frequent in IR-lacking Ulvales plastomes. Gene clusters show a staggered distribution pattern on two strands in *Ulva* plastomes. Our new findings have deepened our understanding of the evolutionary trend of the plastomes in ecologically important *Ulva* seaweeds.

## Data availability statement

The datasets presented in this study can be found in online repositories. The names of the repository/repositories and accession number(s) can be found below: GenBank OL684341, OL684342, and OP985129-OP985133.

## Author contributions

FL designed the study. FL, NC, HW, JL, JW and FQ performed the experiments. FL and HW performed the analysis. FL wrote the manuscript. All authors contributed to the article and approved the submitted version.
