# Supplementary material for: Novel insights into chloroplast genome evolution in the green macroalgal genus Ulva (Ulvophyceae, Chlorophyta)
Source: Front Plant Sci. 2023 Apr 18;14:1126175. doi: 10.3389/fpls.2023.1126175 (PMC10151680; doi:10.3389/fpls.2023.1126175)
Supplement: Supplementary file 1 [file DataSheet_1.pdf]

Fig. S1 Insertion site, size and type of introns detected in 40 *Ulva* plastomes.

Introns	*Upr1*	*Upr2*	*Upr3*	*Upr4*	*Upr5*	*Uli*	*Uto1*	*Uto2*
atpA -492					1171			
atpB -537								
atpB -627								
atpB -696					2400		2370	2370
atpI -256								
infA -62	616	616	616	616	616	611	574	574
petB -23								
petB -69					2257		2205	2215
petB -169								
petB -277							2442	2442
petB -528	1250	1250	1250	1249	1250		1272	1272
petD -87								
psaA -1104								
psaA -1605							1096	1096
psaB -1050	1147	1147	1147	1147	1147			
psbA -179								
psbA -750							982	982
psbB -489	1011	1011	1011	1011	1003			
psbB -600								
psbB -772								
psbB -1022								
psbB- 1352								
psbC -496								
psbC -708							982	982
psbC -882								
psbD -740					1033		1042	
psbD -1034							929	1082
rnl- 1893*a*								
rnl- 1893*b*							1004	1004
rnl -2225							953	953
rnl -2463							1012	1012
rnl -2556								
rns -476								
rns -499								

Introns	*Uca*	*Uar1*	*Uar2*	*Ugi*	*Ula1*	*Ula2*	*Uoh*	*Ulc1*
atpA -492	1183			1133				
atpB -537								
atpB -627			2242	2237			2222	
atpB -696	2375	2371	2371	2387			2348	2364
atpI -256				2252				
infA -62	664	650	650	563	580	580	575	571
petB -23								
petB -69				1826	2217	2208	2222	2187
petB -169								
petB -277								
petB -528				1275	1290	1290		
petD -87				2427				
psaA -1104								
psaA -1605								
psaB -1050				1089	1113	1114	1113	
psbA -179								
psbA -750								
psbB -489				624				
psbB -600				1295				
psbB -772								
psbB -1022	969							
psbB- 1352								
psbC -496								
psbC -708								
psbC -882	932							
psbD -740	1036				1005	1005		
psbD -1034								
rnl- 1893*a*					770	770	763	763
rnl- 1893*b*								
rnl -2225	950						953	953
rnl -2463								
rnl -2556				799				
rns -476								
rns -499								

Introns	*Ulc2*	*Ulc3*	*Ulc4*	*Usp2*	*Ume*	*Usp1*	*Ute*	*Usp3*
atpA -492					1155	1138		
atpB -537					2355			
atpB -627	2203	2205						
atpB -696	2364	2364	2364		2376			
atpI -256								
infA -62	571	571	571	578	573	569	586	571
petB -23					2317			
petB -69	2232	2232	2232			2211		
petB -169								
petB -277	2443				2473			
petB -528								
petD -87								
psaA -1104					1237			
psaA -1605								
psaB -1050					1133			
psbA -179					695			
psbA -750								
psbB -489							360	
psbB -600		1299			1224	1306	1306	
psbB -772					407	351	342	
psbB -1022							952	
psbB- 1352		940			954			
psbC -496								
psbC -708		988						
psbC -882		927						
psbD -740		1056			1075			
psbD -1034								
rnl- 1893*a*	763	763	763			767		
rnl- 1893*b*					1012			
rnl -2225	953	953	953	953	950			950
rnl -2463								
rnl -2556								
rns -476					1008			1004
rns -499							1809	369

Introns	*Usp3* (*Upr*)	*Usp3* (*Ume*)	*Uco1*	*Uco2*	*Uco3*	*Uco4*	*Uco5*	*Uco6*
atpA -492			1173		1173	1173	1173	1173
atpB -537								
atpB -627			2232	2223	2224	2232		2224
atpB -696			2373			2369		
atpI -256								
infA -62	571	571	739	728	761	739	761	761
petB -23								
petB -69			2268		2227	2268		2213
petB -169								
petB -277								
petB -528			1265			1265		
petD -87				2444	2420			
psaA -1104								
psaA -1605								
psaB -1050								
psbA -179								
psbA -750								
psbB -489								
psbB -600								
psbB -772								
psbB -1022								
psbB- 1352								
psbC -496						2441		
psbC -708								
psbC -882								
psbD -740			1036			1036		
psbD -1034								
rnl- 1893*a*			763		763	763	763	763
rnl- 1893*b*								
rnl -2225	950	950	976		975	976	975	975
rnl -2463								
rnl -2556			752			752		
rns -476	1004	1010						
rns -499	369	369						

Introns	*Uco7*	*Uin*	*Uri1*	*Uri2*	*Ufe*	*Uau1*	*Uau2*	*Uau3*
atpA -492	1173	1162	1200	1186		1162	1168	1168
atpB -537								
atpB -627	2224		2211	2211	2250	2235		
atpB -696			2394	2370				
atpI -256								
infA -62	761	596	569	569	556	561	561	561
petB -23			2315	2315				
petB -69	2227	2216					2235	2235
petB -169			2459	2459				
petB -277			2442	2442				
petB -528			1268	1267				
petD -87	2420							
psaA -1104			1239	1239				
psaA -1605								
psaB -1050			1099	1099		1112	1119	1118
psbA -179								
psbA -750						291		
psbB -489		1007						
psbB -600		1375	1300	1300				
psbB -772								
psbB -1022		957						
psbB- 1352								
psbC -496								
psbC -708		993						
psbC -882			923	923				
psbD -740			1055	1055				
psbD -1034								
rnl- 1893*a*	763	769			767	771	767	767
rnl- 1893*b*								
rnl -2225	975	973	1057	985				
rnl -2463			1013	1013				
rnl -2556			742	742				
rns -476								
rns -499		1774						

- group IB (complete)
- group I (derived A)
- group I (derived B1)
- group I (derived B2)
- group IA
- group IA3
- group I (unknown)
- group IIA
- group IIB
- group II (derived)
