# Supplementary material for: Novel insights into chloroplast genome evolution in the green macroalgal genus Ulva (Ulvophyceae, Chlorophyta)
Source: Front Plant Sci. 2023 Apr 18;14:1126175. doi: 10.3389/fpls.2023.1126175 (PMC10151680; doi:10.3389/fpls.2023.1126175)

Fig. S3 Synteny comparison of plastomes from different *Ulva* lineages using Mauve software. Rectangular blocks of the same color indicate collinear regions of sequences.

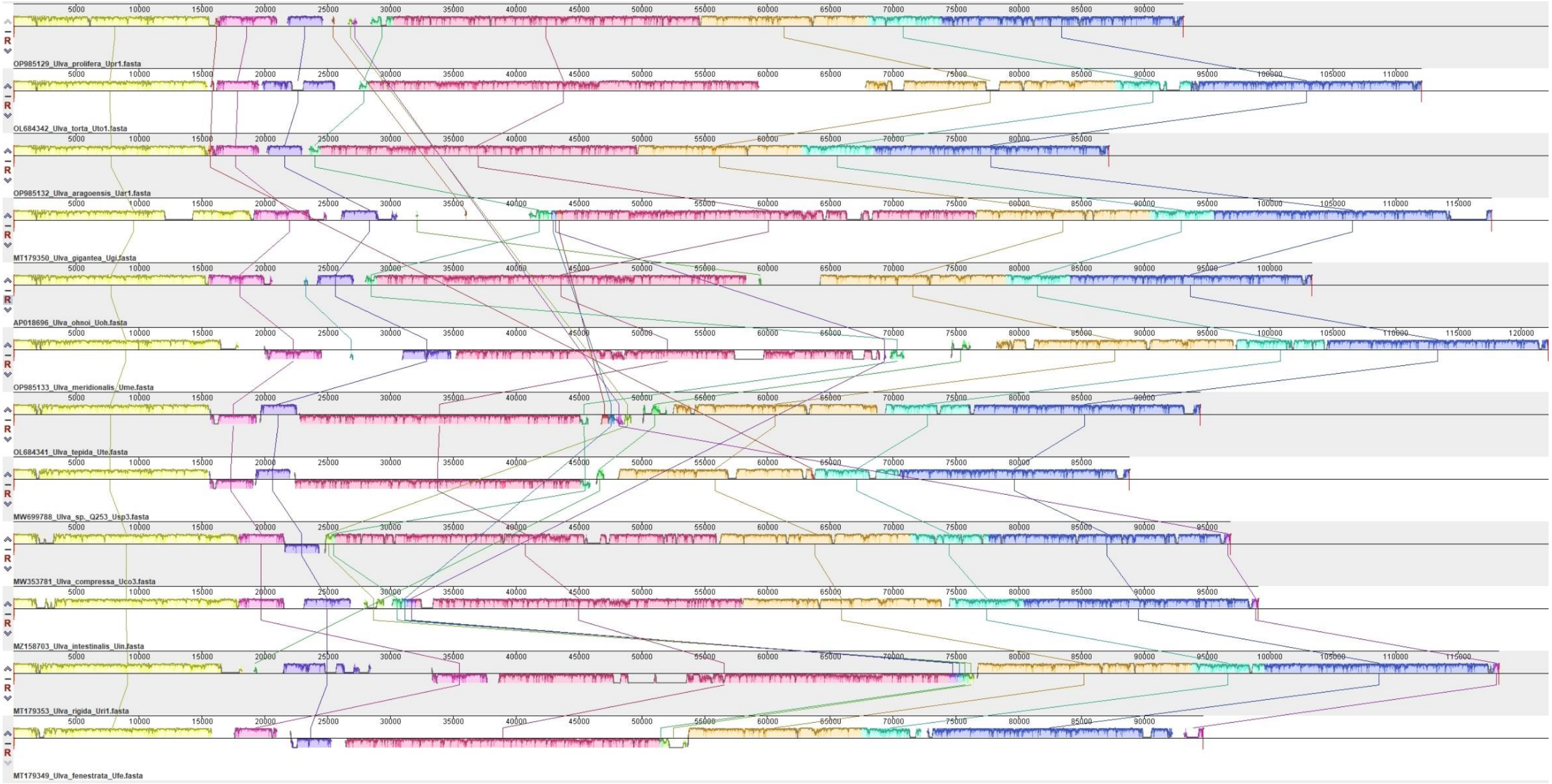

Supplement: Supplementary file 3 [file DataSheet_3.pdf]
