# Supplementary material for: Novel insights into chloroplast genome evolution in the green macroalgal genus Ulva (Ulvophyceae, Chlorophyta)
Source: Front Plant Sci. 2023 Apr 18;14:1126175. doi: 10.3389/fpls.2023.1126175 (PMC10151680; doi:10.3389/fpls.2023.1126175)
Supplement: Supplementary file 4 [file DataSheet_4.pdf]

Table S1. The circular complete chloroplast genomes sequenced in Ulvophyceae.

Order	Species	Accession number	Size (bp)
Ulvales (42)	*Ulva prolifera*	OP985129	93,066
	Ulva prolifera	OP985130	93,066
	Ulva prolifera	OP985131	93,072
	Ulva prolifera	KX342867	93,066
	Ulva prolifera	MZ571508	99,724
	Ulva linza	KX058323	86,726
	Ulva torta	OL684342	112,034
	Ulva torta	MZ703011	105,423
	Ulva californica	MZ561475	92,126
	Ulva aragoënsis	OP985132	87,172
	Ulva aragoënsis (*Ulva flexuosa**)	KX579943	89,414
	Ulva gigantea	MT179350	117,606
	Ulva lactuca (syn. *Ulva fasciata*)	KT882614	96,005
	Ulva lactuca	MH730972	95,997
	Ulva ohnoi	AP018696	103,313
	Ulva lacinulata (*Ulva laetevirens**)	MT179351	103,444
	Ulva lacinulata	MW543061	107,242
	Ulva lacinulata (*Ulva laetevirens**)	MW531676	110,889
	Ulva lacinulata (*Ulva rigida**)	MN389525	103,523
	Ulva sp. A AF-2021 (*Ulva rigida**)	MT179352	96,673
	Ulva meridionalis	OP985133	122,172
	Ulva sp. UNA00071828	KP720616	99,983
	Ulva tepida	OL684341	94,449
	Ulva sp. Q253	MW699788	88,801

	Ulva sp. (*Ulva prolifera**)	MN853879	88,801
	Ulva sp. (*Ulva meridionalis**)	MN889540	88,653
	Ulva compressa	MW548841	114,291
	Ulva compressa	MW344287	91,189
	Ulva compressa	MW353781	96,824
	Ulva compressa (syn. *Ulva mutabilis*)	MK069584	119,866
	Ulva compressa	MK069585	>89,164
	Ulva compressa	MT916929	94,226
	Ulva compressa	KX595275	96,808
	Ulva intestinalis	MZ158703	99,041
	Ulva rigida (*Ulva rotundata**)	MT179353	118,206
	Ulva rigida	MW543060	117,995
	Ulva fenestrata	MT179349	94,654
	Ulva australis (syn. *Ulva pertusa*)	MN853875	104,380
	Ulva australis	LC507117	102,899
	Ulva australis	MT179348	99,820
	Blidingia minima	MK408749	170,562
	Blidingia minima	MT948112	170,656
Sykidiales (1)	*Pseudoneochloris marina*	KY407657	134,753
Ulotrichales (8)	*Capsosiphon fulvescens*	MG727869	111,561
	Capsosiphon fulvescens	MH790906	111,563
	Sarcinofilum mucosum	KY407656	227,181
	Hazenia capsulata	KY407661	189,599
	Pseudendoclonium akinetum	KY407658	195,867
	Gloeotilopsis planctonica	KX306824	221,431
	Rhexinema sarcinoideum	KX306821	262,888

	Gloeotilopsis sterilis	KM462877	132,626
Oltmannsiellopsidales (2)	*Oltmannsiellopsis viridis*	DQ291132	151,933
	Dangemannia microcystis	KY407660	166,355
Bryopsidales (48)	*Codium fragile*	MN853876	83,788
	Codium fragile	MN733705	83,422
	Codium arenicola	KY819066	83,933
	Codium simulans	KT946603	91,509
	Codium arabicum	MH591107	79,309
	Bryopsis hypnoides	GQ892829	153,429
	Bryopsis plumosa	LN810504	106,859
	Bryopsis sp.	KY819063	100,655
	Lambia antarctica	KU059765	104,098
	Caulerpa serrulata	MK792749	177,848
	Caulerpa sertularioides f. longipes	MK792750	133,626
	Caulerpa manorensis	KY819068	140,597
	Caulerpa okamurae	KX809677	148,274
	Caulerpa racemosa	KT946602	176,522
	Caulerpa cupressoides	MG797569	130,895
	Caulerpa lentillifera	MG753774	119,402
	Caulerpa lentillifera	MN201587	123,255
	Caulerpa lentillifera	MT271684	126,969
	Caulerpa cliftonii	KX808498	131,135
	Caulerpa verticillata	MH591106	148,111
	Caulerpa ashmeadii	MH745228	135,722
	Caulerpa taxifolia	OK139149	143,328
	Ostreobium sp.	KY509314	80,584

	Ostreobium sp.	KU979013	81,997
	Ostreobium quekettii	OK189523	80,307
	Ostreobium quekettii	OK189524	79,957
	Ostreobium quekettii	OK189525	80,030
	Ostreobium quekettii	OK189526	79,921
	Ostreobium quekettii	OK189527	83,152
	Ostreobium quekettii	OK189528	87,754
	Ostreobium quekettii	OK189529	81,111
	Ostreobium quekettii	OK189530	80,761
	Ostreobium quekettii	OK189531	80,614
	Pedobesia claviformis	MH591108	75,391
	Rhipilia penicilloides	KY819065	105,774
	Rhipiliopsis peltata	MH591110	114,851
	Pseudocodium devriesii	MH591109	94,006
	Callipsygma wilsonis	MH591105	74,525
	Chlorodesmis fastigiata	KY819064	88,592
	Tydemania expeditiones	LN810505	105,200
	Udotea sp.	MH591113	90,749
	Udotea argentea	MH591111	91,555
	Udotea flabellum	MH591112	100,743
	Boodleopsis pusilla	MH591103	107,336
	Boodleopsis sp.	MH591104	75,962
	Boodleopsis sp.	MH591102	88,034
	Derbesia sp.	KX808497	115,765
	Halimeda opuntia	ON584177	106,249
Ignatiales (2)	*Ignatius tetrasporus*	KY407659	239,387

	Pseudocharacium americanum	KY407658	239,448
Trentepohliales (7)	*Trentepohlia odorata*	MK580484	399,372
	Trentepohlia sp.	MZ334625	216,308
	Trentepohlia sp.	MZ334626	408,697
	Cephaleuros virescens	MW822747	314,936
	Cephaleuros tumidae-setae	MW822748	282,795
	Cephaleuros karstenni	MZ334628	371,192
	Cephaleuros parasiticus	MZ334627	266,729
