# Supplementary material for: Novel insights into chloroplast genome evolution in the green macroalgal genus Ulva (Ulvophyceae, Chlorophyta)
Source: Front Plant Sci. 2023 Apr 18;14:1126175. doi: 10.3389/fpls.2023.1126175 (PMC10151680; doi:10.3389/fpls.2023.1126175)
Supplement: Supplementary file 5 [file DataSheet_5.pdf]

Table S2 Comparison of size and GC composition in different regions of *Ulva* plastomes.

Species	Plastomes		100 canonical genes		71 PCGs		3 rRNAs	
	Size (bp)	GC (%)	Size (bp)	GC (%)	Size (bp)	GC (%)	Size (bp)	GC (%)
Upr1	93066	24.78	72401	27.26	65980	25.34	4455	44.92
Upr2	93066	24.78	72386	27.26	65965	25.35	4455	44.92
Upr3	93072	24.78	72322	27.23	65901	25.31	4455	44.92
Upr4	93066	24.78	72393	27.26	65973	25.34	4454	44.90
Upr5	99724	25.28	72385	27.26	65964	25.34	4455	44.94
Uli	86726	24.79	72268	27.25	65847	25.33	4455	44.92
Uto1	112034	24.89	71535	26.95	65127	24.96	4442	45.20
Uto2	105423	25.24	71465	26.95	65057	24.96	4442	45.20
Uca	92126	24.71	71532	26.95	65124	24.97	4442	45.09
Uar1	87172	24.68	71548	26.95	65140	24.97	4442	45.09
Uar2	89414	24.97	71548	26.95	65140	24.97	4442	45.09
Ugi	117606	25.73	71544	26.93	65122	24.95	4456	45.00
Ula1	96005	24.87	71385	27.14	64972	25.17	4447	45.00
Ula2	95997	24.87	71376	27.16	64963	25.21	4447	45.00
Uoh	103313	25.44	71363	27.19	64954	25.23	4443	45.04
Ulc1	103444	25.40	71382	27.14	64972	25.16	4444	45.16
Ulc2	107242	25.82	71366	27.16	64957	25.19	4443	45.17
Ulc3	110889	25.63	71403	27.15	64993	25.17	4444	45.16
Ulc4	103523	25.40	71403	27.16	64993	25.18	4444	45.16
Usp2	96673	24.57	71412	27.15	65003	25.18	4443	45.13
Ume1	122172	24.86	71504	26.96	65089	24.97	4449	45.07
Usp1	99983	25.30	71407	26.77	64993	24.78	4450	44.88
Ute	94449	24.49	71421	26.98	65014	24.99	4441	45.08
Usp3	88801	23.89	71357	26.53	64945	24.50	4446	45.01
Usp3	88801	23.89	71357	26.53	64945	24.50	4446	45.01
Usp3	88653	23.91	71244	26.56	64833	24.53	4445	45.02
Uco1	114291	26.23	72347	27.57	65935	25.67	4446	45.14
Uco2	91189	25.86	72455	27.68	66040	25.79	4449	45.11
Uco3	96824	26.17	72459	27.69	66046	25.80	4447	45.15
Uco4	119866	26.24	72341	27.56	65929	25.65	4446	45.14
Uco5	89164	26.25	—	—	—	—	4447	45.15
Uco6	94226	25.80	72327	27.60	65914	25.69	4447	45.15
Uco7	96808	26.18	72465	27.71	66052	25.82	4447	45.15
Uin	99041	24.97	71557	27.17	65146	25.22	4445	44.95
Uri1	118206	26.12	71445	27.12	65026	25.17	4453	44.76
Uri2	117995	26.13	71445	27.13	65026	25.18	4453	44.76
Ufe	94654	25.27	71620	27.20	65209	25.27	4445	44.86
Uau1	104380	25.66	71582	27.21	65161	25.29	4455	44.67
Uau2	102899	25.33	71614	27.25	65182	25.33	4465	44.61
Uau3	99820	25.21	71571	27.23	65140	25.30	4465	44.61

**Species**	**26 tRNAs**		***minD* region**		***trnR3***		**Intron region**	
	**Size (bp)**	**GC (%)**	**Size (bp)**	**GC (%)**	**Size (bp)**	**GC (%)**	**Size (bp)**	**GC (%)**
Upr1	1966	51.58	—	—	72	33.33	4024	23.53
Upr2	1966	51.58	—	—	72	33.33	4024	23.53
Upr3	1966	51.58	—	—	72	33.33	4024	23.53
Upr4	1966	51.58	—	—	72	33.33	4023	23.54
Upr5	1966	51.58	—	—	77	33.77	10877	28.80
Uli	1966	51.53	—	—	—	—	611	22.59
Uto1	1966	51.58	911	18.33	72	34.72	16863	29.62
Uto2	1966	51.58	911	18.33	—	—	15984	29.85
Uca	1966	51.58	926	24.41	—	—	8109	28.23
Uar1	1966	51.63	921	24.54	—	—	3021	33.43
Uar2	1966	51.58	921	24.54	—	—	5263	34.51
Ugi	1966	51.53	—	—	72	38.89	17907	31.85
Ula1	1966	51.58	—	—	—	—	6975	27.89
Ula2	1966	51.58	—	—	—	—	6967	27.89
Uoh	1966	51.58	—	—	—	—	10196	32.27
Ulc1	1966	51.68	—	—	72	34.72	6838	33.24
Ulc2	1966	51.68	—	—	72	34.72	11529	34.52
Ulc3	1966	51.68	—	—	72	34.72	14298	30.94
Ulc4	1966	51.68	—	—	72	34.72	6883	33.27
Usp2	1966	51.58	—	—	72	34.72	1531	24.82
Ume1	1966	51.73	—	—	72	36.11	20944	28.87
Usp1	1964	51.63	—	—	—	—	6342	28.35
Ute	1966	51.88	—	—	—	—	5355	24.20
Usp3	1966	51.78	—	—	—	—	2894	25.40
Usp3	1966	51.78	—	—	—	—	2894	25.40
Usp3	1966	51.78	—	—	—	—	2900	25.38
Uco1	1966	51.63	—	—	—	—	13577	30.63
Uco2	1966	51.73	—	—	—	—	5395	34.72
Uco3	1966	51.68	—	—	—	—	10543	33.01
Uco4	1966	51.68	—	—	—	—	16014	31.58
Uco5	1966	51.68	—	—	—	—	3672	26.31
Uco6	1966	51.68	—	—	—	—	8109	31.51
Uco7	1966	51.68	—	—	—	—	10543	33.01
Uin	1966	51.63	—	—	—	—	11822	27.30
Uri1	1966	51.63	—	—	72	36.11	23286	30.13
Uri2	1966	51.63	—	—	72	36.11	23175	30.15
Ufe	1966	51.32	—	—	—	—	3573	34.28
Uau1	1966	51.63	—	—	—	—	6132	28.93
Uau2	1967	51.50	—	—	—	—	5850	28.51
Uau3	1966	51.63	—	—	—	—	5849	28.53

Species	Specific *orf* region		Non-coding spacer region	
	Size (bp)	GC (%)	Size (bp)	GC (%)
Upr1	1905	25.83	14664	12.68
Upr2	1905	25.83	14679	12.67
Upr3	1905	25.83	14749	12.92
Upr4	1906	25.81	14672	12.75
Upr5	1482	27.26	14903	12.88
Uli	—	—	13847	12.01
Uto1	8153	22.79	14500	10.77
Uto2	4958	25.09	12105	9.65
Uca	—	—	11559	8.38
Uar1	—	—	11682	8.53
Uar2	—	—	11682	8.58
Ugi	6091	29.21	21992	15.85
Ula1	3511	23.75	14134	12.18
Ula2	3511	23.81	14143	12.05
Uoh	5931	23.96	15823	13.72
Ulc1	9340	27.22	15812	13.06
Ulc2	8423	26.96	15852	12.80
Ulc3	9330	27.21	15786	12.97
Ulc4	9337	27.21	15828	12.94
Usp2	6884	26.76	16774	12.66
Ume1	14556	22.90	15096	11.22
Usp1	5419	34.36	16815	14.96
Ute	4387	27.65	13286	10.20
Usp3	1081	18.87	13469	9.95
Usp3	1081	18.87	13469	9.95
Usp3	1081	18.69	13428	9.93
Uco1	11144	30.08	17223	14.65
Uco2	—	—	13339	12.35
Uco3			13822	13.01
Uco4	14265	27.47	17246	14.70
Uco5	—	—	17110	16.56
Uco6	—	—	13790	13.02
Uco7	—	—	13800	12.97
Uin	1397	25.34	14265	11.95
Uri1	8127	27.02	15276	14.81
Uri2	8131	26.93	15172	14.82
Ufe	1146	24.26	18315	16.06
Uau1	4232	28.62	22434	19.25
Uau2	3601	29.71	21834	17.48
Uau3	547	35.83	21853	17.43
