# Supplementary material for: Novel insights into chloroplast genome evolution in the green macroalgal genus Ulva (Ulvophyceae, Chlorophyta)
Source: Front Plant Sci. 2023 Apr 18;14:1126175. doi: 10.3389/fpls.2023.1126175 (PMC10151680; doi:10.3389/fpls.2023.1126175)
Supplement: Supplementary file 6 [file DataSheet_6.pdf]

Table S3 The 11 homologous groups of plastome-specific *orf*s detected in 40 *Ulva* chloroplast genomes.

Groups	Homologous *orf*s *	Locations (intergenic region)	Putative function
1	*orf181 Usp3* (+); *orf181 Usp3*(*Upr*) (+); *orf181 Usp3*(*Ume*) (+); *orf183 Ute* (+); *orf185 Ume* (+); *orf167 Ume* (+)	*psbA-psbB*	Ucp-*orf*, unknown
	orf178 Ugi (+); *orf149 Ulc1-4* (+); *orf149 Usp2* (+);*orf196 Ulc1-4* (-); *orf196 Usp2* (-); *orf169 Uin* (+)	*psbC-psbB*	
	orf149 Uri1,2 (+); *orf179a Uri1,2* (-); *orf179 Ufe* (+)	*psbC-trnM3*	
	orf187 Uto1 (+); *orf153 Uto1* (-); *orf180 Uoh* (-)	*trnT-psbA*	
	orf204 Ume (+); *orf192 Ume* (+)	*psbC-trnS2*	
	orf190 Ume (+)	*trnL2-trnM3*	
2	*orf245 Ulc1,3,4* (-); *orf175 Ula1,2* (+)	*trnT-psbA*	putative bacterial tyrosine-type recombinase/integrase (*tri*)
	orf118-orf102 Ugi (+); *orf590 Uto2* (-); *orf590 Uoh* (-)	*trnM3-psbD*	
	orf618 Uco1 (+); *orf428 Uco4* (+)	*psbD-psbB*	
	orf619 Usp2 (+)	*psbC-psbB*	
	orf67 Uau2 (-)	*trnM1-trnE*	
	orf154 Uau1 (+)	*trnW-psaJ*	
3	*orf88 Ulc1,3,4* (-); *orf90 Ulc2* (-); *orf90 Usp2* (-)	*trnM3-psbD*	NAD-dependent DNA ligase (*lig*)
	orf601 Usp1 (+)	*psbA-psbB*	
	orf191 Uco1,4 (+)	*trnT-psbA*	
4	*orf1450 Uco1* (-); *orf218-orf894 Uco4* (-); *orf144 Ula1,2* (+); *orf182 Uoh* (-)	*trnT-psbA*	phage/plasmid DNA primase
	orf205-orf434 Uri1 (-); *orf205-orf177-orf197 Uri2* (-)	*trnL2-psbD*	
	orf100 Ufe (-)	*chlI-rps32*	
5	*orf493 Upr5* (-); *orf134-orf175-orf181 Upr1,2,3* (-);	*psbC-psbB*	Unknown

	orf134-orf175-orf174 Upr4 (-)		
	orf137 Ume (-)	*psbC-trnS2*	
6	*orf476 Ulc1* (+); *orf384 Ulc2* (+); *orf500 Ulc3* (+); *orf112 Usp2* (+); *orf415 Ula1,2* (-)	*trnT-psbA*	Unknown
	orf201 Uto2 (+); *orf147 Uoh* (+)	*trnM3-psbD*	
	orf156 Uco1,4 (-)	*psbD-psbB*	
	orf117 Uau2 (+)	*trnM1-trnE*	
	orf154 Uau1 (+)	*trnW-psaJ*	
7	*orf134 Ulc1-4* (+); *orf132 Ula1,2* (-)	*trnT-psbA*	Unknown
8	*orf128 Ulc1,3,4* (+); *orf179 Ulc2* (+)	*trnT-psbA*	Unknown
	orf219 Uco4 (+)	*psbD-psbB*	
	orf140 Uto1 (+)	*psbC-psbB*	
9	*orf683 Ume* (+); *orf646 Ute* (+)	*psbA-psbB*	Unknown
	orf621 Uto1 (-); *orf228 Uto2* (-)	*trnT-psbA*	
	orf179b Uri2 (+)	*psbC-trnM3*	
	orf514 Ume (+)	*trnL2-trnM3*	
10	*orf707 Uco1* (-); *orf345-orf119 Uco4* (-); *orf191 Uoh* (-)	*trnT-psbA*	Unknown
11	*orf119 Usp3* (+); *orf119 Usp3*(*Upr*) (+); *orf119 Usp3*(*Ume*) (+); *orf136 Ume* (+)	*psbA-psbB*	Unknown

***** The *orf*s from different intergenic regions share homologous sequences, while their sizes vary greatly due to multiple mutations.
