# Supplementary material for: Novel insights into chloroplast genome evolution in the green macroalgal genus Ulva (Ulvophyceae, Chlorophyta)
Source: Front Plant Sci. 2023 Apr 18;14:1126175. doi: 10.3389/fpls.2023.1126175 (PMC10151680; doi:10.3389/fpls.2023.1126175)

Fig. S2 Genome organization and gene order of *Ulva* plastomes. Thick lines with different colors on the left represented gene blocks. Gene types were shown as filled boxes in different colors.

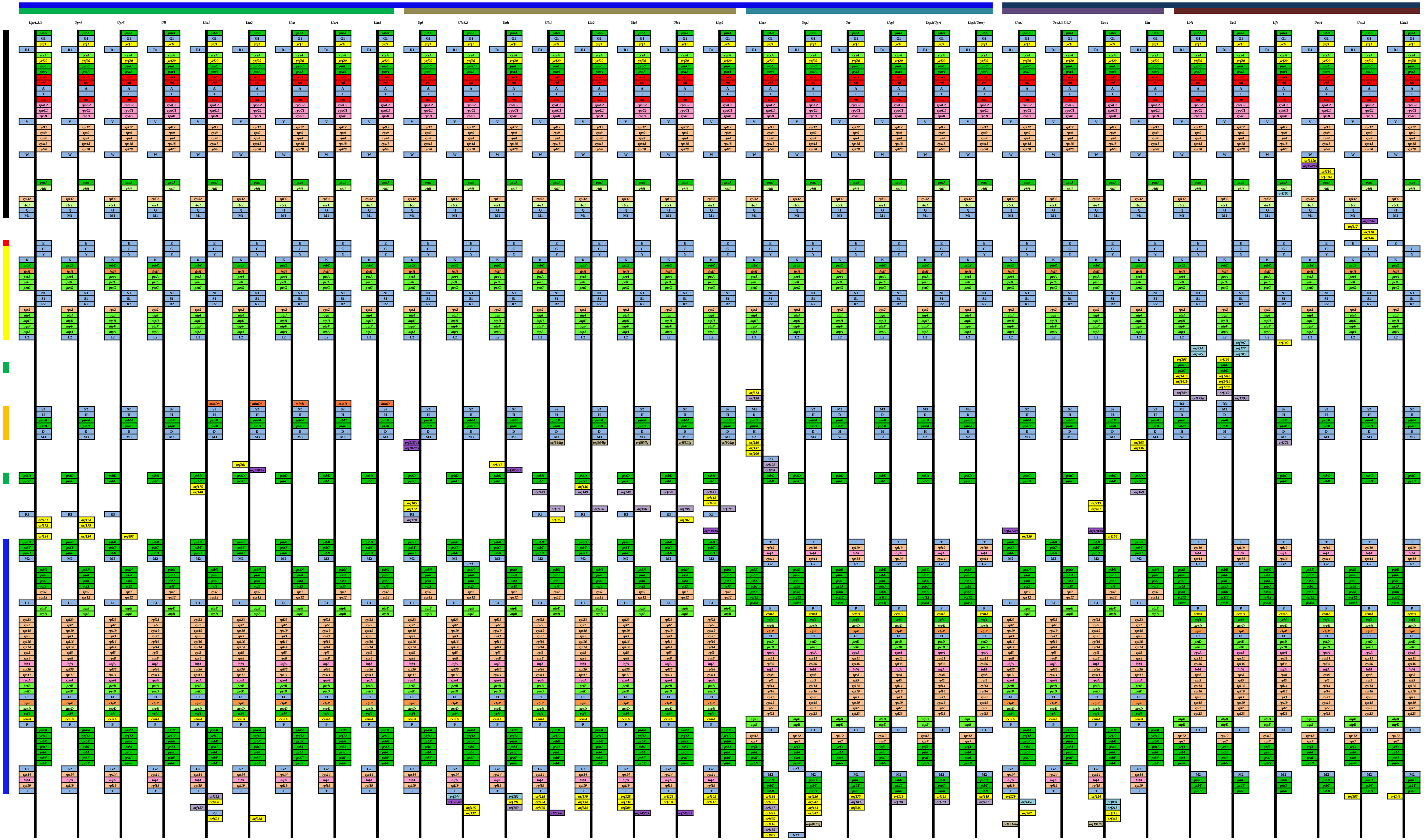

Supplement: Supplementary file 2 [file DataSheet_2.pdf]
